# EMamba: A MoE-enhanced state space network for robust steel surface defect detection

**DOI:** 10.1371/journal.pone.0358866

**Published:** 2026-09-25

**Authors:** Haiyan Yang, Peng Wang, Yangyang Liu

**Affiliations:** Technological Institute of Materials & Energy Science, Xijing University, Xi’an, China; Hohai University, CHINA

## Abstract

Steel surface defect detection is a critical component of intelligent manufacturing and industrial visual quality control. However, due to large variations in defect scale, elongated morphologies, strong directional continuity, and high inter-class visual similarity, existing detection methods struggle to achieve a desirable balance among global structural modeling, semantically adaptive representation, and computational efficiency. To address these challenges, this paper proposes an efficient steel surface defect detection framework, termed EMamba. By integrating state space modeling, conditional expert computation, and a hybrid multi-branch architectural design, the proposed framework systematically enhances detection performance in complex industrial scenarios. Specifically, we introduce a two-dimensional state space modeling mechanism and propose a Convolutional State Space Block (CSSBlock), which models long-range spatial dependencies and directional continuity with linear computational complexity. At the high-level semantic stage of the backbone network, an Efficient Sparse Mixture-of-Experts module (ES-MoE) is incorporated. Through a conditional Top-k routing mechanism, ES-MoE enhances the diversity and discriminability of feature representations. Furthermore, we propose a Mixed Star Network (MSN), which selectively configures high-order feature modeling modules in a hierarchy-aware manner within a multi-branch structure, achieving a fine-grained trade-off between representational capacity and computational efficiency. Building upon these components, a decoupled detection head is adopted to construct the unified EMamba detection framework. Extensive experiments on multiple steel surface defect datasets demonstrate that EMamba achieves competitive detection accuracy, structural defect perception capability, and inference efficiency compared with representative existing methods. These results indicate the effectiveness and practicality of the proposed hybrid modeling paradigm for steel surface defect detection.

## 1. Introduction

**Fig 1:**
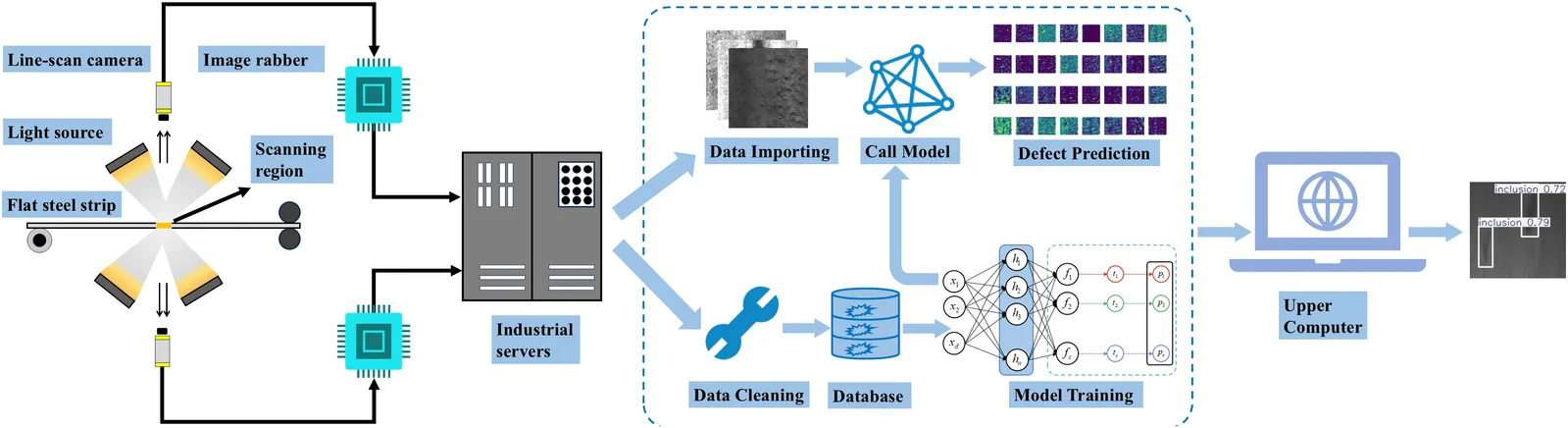
Overall pipeline of the steel surface defect detection system.

Steel surface defect detection is a critical component of intelligent manufacturing and industrial visual quality control, with broad applications in rolling production, coating inspection, and structural safety assessment [1,2]. As illustrated in Fig 1, a typical steel surface defect detection system consists of industrial cameras, edge computing chips, a host-side database, and a detection result visualization module.

The industrial cameras capture surface images of steel materials in real time and transmit them to the edge computing chip, where the proposed detection model performs online analysis of defect categories and locations. The detection results, along with intermediate feature information, are simultaneously uploaded to the host database for storage and management. Through a visualization interface, the defect detection outcomes are presented to operators, thereby supporting subsequent quality evaluation and production decision-making.

**Fig 2:**
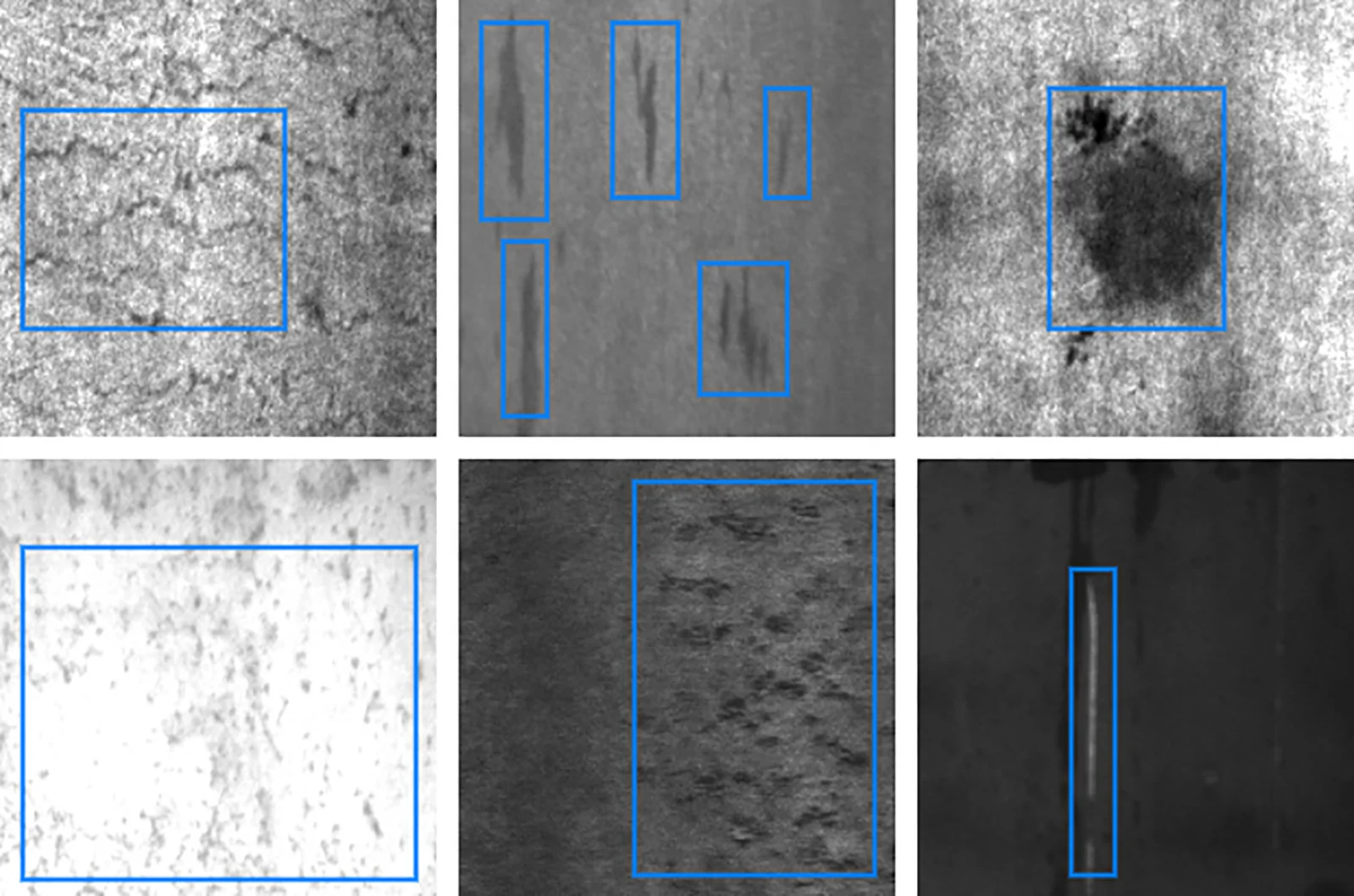
Six defect categories in the NEU-DET dataset.

As illustrated in Fig 2, unlike object detection in natural images, steel surface defects typically exhibit large scale variations, elongated morphologies, strong directional continuity, and high inter-class visual similarity. These characteristics impose more stringent requirements on detection models in terms of fine-grained local texture representation, long-range structural modeling, and high-level semantic discrimination capability.

In recent years, research on steel surface defect detection has primarily focused on lightweight detection frameworks, multi-scale feature fusion, and enhanced small-object detection capabilities [3–8]. Improvement strategies based on one-stage detectors such as YOLO have introduced multi-scale feature enhancement modules and attention mechanisms, effectively improving detection accuracy under complex backgrounds and high inter-class similarity, and demonstrating strong performance across multiple steel defect datasets [9]. To further balance detection accuracy and computational efficiency, some studies have incorporated Cross Stage Partial (CSP) networks, depthwise separable convolutions (DSConv), and Bidirectional Feature Pyramid Networks (BiFPN) into backbone and neck architectures, strengthening cross-scale semantic interaction while maintaining high inference speed [10]. In addition, several works have redesigned overall network architectures for industrial scenarios, proposing lightweight detection models equipped with feature enhancement fusion modules or context-aware mechanisms to improve robustness under complex backgrounds and low-contrast defect conditions [11,12].

Despite these advances in local texture modeling and multi-scale fusion, most existing approaches still rely on local convolution operations or static feature transformations, limiting their ability to model the long-range continuous structures and directional consistency patterns that are prevalent in steel surface defects. Moreover, the introduction of enhancement modules within multi-branch architectures often lacks hierarchy-aware and selective configuration, leading to computational redundancy. These issues remain challenging in high-resolution industrial inspection scenarios.

To address the above limitations, this paper proposes an efficient detection framework, termed EMamba, for steel surface defect detection. The goal is to enhance global modeling capability, semantic adaptability, and structural modeling efficiency while preserving practical deployability. Specifically, a CSSBlock is introduced into the backbone network to model long-range spatial dependencies with linear complexity, thereby improving sensitivity to elongated cracks and directionally continuous structural defects. At the high-level semantic stage, an ES-MoE is further integrated, leveraging a conditional expert routing mechanism to enhance the diversity and discriminability of feature representations. Building upon these components, a MSN is proposed to hierarchically and selectively configure high-order feature modeling modules within a multi-branch architecture, achieving a more favorable trade-off between representational capacity and computational efficiency. Finally, a decoupled detection head is adopted to construct the complete detection framework, enabling precise localization and recognition of complex steel surface defects.

In summary, the main contributions of this paper are as follows:

1) We introduce a two-dimensional state space modeling mechanism into the classical CSP framework and propose a CSSBlock. While maintaining linear computational complexity and strong engineering compatibility, CSSBlock effectively enhances the network’s ability to model long-range spatial dependencies and directionally continuous structures, making it particularly suitable for representing elongated cracks and structural defects.
2) To improve the conditional adaptive modeling capability of high-level semantic features, we integrate an ES-MoE. Through a Top-K sparse routing mechanism, ES-MoE enables input-dependent expert selection and semantic reorganization while significantly controlling computational overhead.
3) To address the lack of hierarchical differentiation in module configuration within multi-branch architectures, we propose a MSN, achieving a fine-grained balance between high-order nonlinear interaction capability and computational efficiency. This hybrid design avoids the redundant computation caused by full-scale module replacement and provides a general and tunable structural paradigm for enhanced multi-branch networks.
4) Based on the above module and architectural designs, we further construct a unified steel surface defect detection framework, termed EMamba. In this framework, CSSBlock serves as the fundamental feature modeling unit, ES-MoE is introduced at the high-level semantic stage for conditional expert modeling, and MSN enables efficient feature interaction within the multi-branch structure. While maintaining relatively low computational complexity, EMamba significantly improves detection performance for complex structural defects and highly similar defect categories, providing systematic validation of state space models and conditional computation mechanisms in steel defect detection.

## 2. Related work

### 2.1. General object detection

General object detection is a fundamental research topic in computer vision, aiming to simultaneously achieve precise localization and accurate category recognition in natural images. With the advancement of deep convolutional neural networks, detection frameworks have gradually evolved from traditional two-stage paradigms to more efficient one-stage designs. Representative two-stage detectors, such as the R-CNN family, are exemplified by Faster R-CNN [13], which employs a Region Proposal Network to generate candidate regions followed by separate classification and regression, achieving strong detection accuracy. However, such methods typically incur substantial computational overhead, limiting their applicability in real-time scenarios.

In contrast, one-stage detectors perform dense predictions directly on feature maps, offering significant advantages in inference speed and deployment efficiency. Representative approaches include the YOLO series [14–16] and SSD [17], which enable end-to-end efficient inference while maintaining competitive accuracy, making them widely adopted in real-time detection tasks. In recent years, anchor-free detection paradigms have gained increasing attention. Methods such as CenterNet [18], FCOS [19], YOLOX [20], and RTMDet [21] simplify bounding box modeling and further enhance both performance and engineering practicality. With the continuous evolution of the YOLO family, an effective balance among model lightweight design, detection accuracy, and inference speed has been achieved [22–27], leading to extensive applications in industrial visual inspection.

Meanwhile, the rapid development of Vision Transformers has also driven significant progress in attention-based object detection methods. DETR was the first to introduce the Transformer architecture into object detection, leveraging an end-to-end set prediction mechanism to explicitly model relationships between objects and global context [28]. Building upon this foundation, subsequent methods such as Deformable DETR, DINO, RT-DETR, and DEIM incorporate deformable attention, improved query design, and efficient training strategies to alleviate the slow convergence issue of the original DETR and substantially enhance detection performance [29–33], promoting a paradigm shift from traditional anchor-based designs to query-based frameworks.

Despite the impressive performance of these general-purpose object detection methods on standard natural image benchmarks, they exhibit notable limitations in steel surface defect detection scenarios. First, steel defects often present elongated, continuous structures with strong directional consistency. Purely local spatial modeling based on convolution is insufficient to capture their long-range spatial correlations, leading to limited sensitivity to structural defects such as cracks and scratches. Second, most existing multi-scale feature fusion strategies adopt static fusion mechanisms, treating features from different levels uniformly without selectively enhancing critical semantic stages. This limits the ability to fully exploit discriminative high-order representations while maintaining computational efficiency.

Moreover, mainstream detection frameworks generally rely on static parameter-sharing mechanisms in high-level semantic stages, overlooking the substantial appearance variations of steel defects under different material properties, illumination conditions, and manufacturing processes. This restricts the model’s capability for conditional adaptive modeling. Finally, conventional coupled detection heads may introduce optimization conflicts when jointly handling classification and localization tasks, particularly under ambiguous defect boundaries or small object scales, thereby affecting detection stability and accuracy.

Based on these observations, steel surface defect detection urgently requires a detection framework that preserves computational efficiency and practical deployability while simultaneously possessing long-range structural modeling capability, conditional semantic representation ability, and hierarchy-aware feature fusion mechanisms, precisely the core design motivation of the proposed method.

### 2.2. Defect detection

Industrial surface defect detection is a critical component of intelligent manufacturing and quality control, aiming to achieve precise localization and recognition of tiny, low-contrast, and morphologically diverse defects under complex backgrounds and strict real-time constraints. In recent years, extensive research has focused on feature enhancement, scale modeling, and lightweight architectural design.

Detection methods based on convolutional feature enhancement improve the representation of subtle defects by enlarging receptive fields or incorporating attention mechanisms. For example, RSTD-YOLOv7 [34] strengthens feature extraction through multiple structured convolutional modules and employs a decoupled detection head to alleviate the conflict between classification and regression. FANet [35] adopts a fused attention mechanism to enhance channel-wise and spatial discriminability on unified-scale features. Although these approaches achieve notable improvements in detection accuracy, their feature modeling primarily remains within spatial-domain convolution and local context aggregation, limiting their ability to capture defects characterized by strong structural consistency or distinctive frequency patterns.

Lightweight and real-time variants of the YOLO family have become mainstream choices for steel defect detection. Numerous studies based on YOLOv5/v7/v8 introduce GhostConv, lightweight backbones, or parameter-free attention mechanisms to reduce model complexity and accelerate inference [36–38]. For instance, SS-YOLO and improved YOLOv8 variants achieve high frame rates and reduced parameter counts on the NEU-DET dataset, demonstrating strong potential for practical deployment. However, these methods largely rely on static feature fusion and unified parameter-sharing mechanisms. When faced with substantial scale variations and significant texture distribution shifts among defects, their representational flexibility remains limited.

Specialized architectural designs targeting small objects and fine-grained defects have also emerged as an important research direction. TD-Net [39] reduces information loss of tiny defects through improved downsampling strategies and introduces cross-layer guidance to mitigate semantic conflicts between shallow and deep features. PMSA-DyTr [40] combines a Transformer encoder with a dynamic gating mechanism to achieve cross-scale semantic alignment and computational adaptivity. Some studies further explore multimodal inputs and knowledge distillation strategies to enhance detection robustness in complex scenarios [41]. Despite their strong performance on specific tasks, these methods often adopt relatively complex network structures with increased computational and modeling costs, limiting their applicability in high-resolution industrial inspection scenarios. Traditional two-stage detectors, such as improved Faster R-CNN-based models [42], enhance discrimination of complex-shaped defects via deformable convolution and background suppression mechanisms; however, their inference efficiency is insufficient to meet real-time requirements in practical production lines.

Although existing steel surface defect detection methods have achieved significant progress in feature enhancement, lightweight design, and small-object modeling, they still face several fundamental challenges: (1) feature modeling predominantly relies on spatial-domain convolution, lacking systematic modeling of defect frequency characteristics and long-range structural dependencies; (2) multi-scale fusion strategies are mainly designed in a static manner, making it difficult to adaptively select and enhance features according to hierarchical semantics; and (3) high-level semantic modeling generally adopts shared parameters, limiting the conditional representation capability for diverse defect patterns.

These limitations indicate that existing approaches still struggle to achieve an effective balance among long-range structural modeling, adaptive feature representation, and computational efficiency. To address these challenges, we propose EMamba, an efficient detection framework that integrates long-range dependency modeling, conditional high-level semantic learning, and hierarchy-aware structural design. By combining these complementary components, EMamba enhances the representation capability of complex structural defects while maintaining practical deployability and computational efficiency.

### 2.3. Mamba modeling

In recent years, State Space Models (SSM) have emerged as a promising alternative to Transformers for long-sequence modeling due to their linear time complexity and efficient inference characteristics. Mamba introduces an input-dependent selective state update mechanism, effectively addressing the limitations of traditional SSM in content awareness and information selection. While maintaining linear complexity, it achieves modeling capacity comparable to, or even surpassing, that of attention mechanisms [43]. Benefiting from its hardware-friendly parallel scan design, Mamba demonstrates significant throughput advantages in ultra-long sequence modeling, offering new possibilities for high-resolution visual representation learning.

Building upon this foundation, researchers have begun exploring the application of Mamba to vision tasks. Vision Mamba (Vim) first employed Mamba as a general-purpose visual backbone, introducing bidirectional state space modeling to capture global visual context, achieving modeling performance comparable to Transformers while preserving computational efficiency [44]. Subsequently, VMamba proposed a two-dimensional Selective Scan (SS2D) mechanism to better adapt state space modeling to 2D visual structures, significantly enhancing multi-directional contextual modeling and demonstrating strong scalability across varying input resolutions in multiple vision tasks [45]. Further improvements in efficiency and expressiveness were introduced by methods such as EfficientViM [46] and EAMamba [47], which refined the Vision Mamba architecture from perspectives including hidden state mixing, scanning strategies, and channel organization, achieving favorable performance-efficiency trade-offs in low-level vision tasks.

Meanwhile, several works have extended Mamba to high-level visual understanding and detection frameworks. MambaVision integrates Mamba and Transformer hierarchically, compensating for the limitations of pure Mamba architectures in high-level semantic modeling and achieving superior performance across classification, detection, and segmentation tasks [48]. In object detection, MambaYOLO pioneered the incorporation of SSM-based modeling units into real-time detection frameworks, validating the feasibility of applying Mamba to detection tasks without large-scale pretraining [49]. Additionally, TransMamba and related works explore knowledge transfer mechanisms between Transformers and Mamba through distillation and cross-modal alignment strategies, providing new pathways for applying SSM to complex visual semantic tasks [50].

Furthermore, recent studies have begun to investigate the impact of scanning path designs on the state-space modeling capability in two-dimensional visual spaces. LocalVMamba introduces a local window-based selective scanning mechanism, which enhances local spatial continuity while maintaining the efficiency of global state-space modeling, effectively alleviating the limitation of conventional scanning strategies in capturing local neighborhood relationships. Compared with approaches relying on fixed 2D unfolding patterns, LocalVMamba places greater emphasis on preserving local structural information in visual features, providing a new scanning strategy for Vision Mamba models that balances local representation and long-range dependency modeling [51]. PlainMamba revisits 2D feature modeling in non-hierarchical Mamba architectures from the perspective of simplifying visual state-space designs. By introducing continuous 2D scanning and direction-aware updating mechanisms, PlainMamba improves visual representation capability while maintaining strong recognition performance with reduced architectural complexity [52]. In addition, Mamba-ND extends state-space modeling beyond 2D image scenarios to multi-dimensional data by exploring more general spatial traversal strategies, enhancing dependencies across different dimensions and providing new insights for state-space modeling in multi-dimensional visual tasks [53].

Although these methods have achieved significant progress in 2D scanning strategies, spatial traversal mechanisms, and visual state-space architecture design, their primary focus remains on developing general-purpose visual backbones or improving the intrinsic spatial modeling capability of Mamba. For steel surface defect detection, defect regions usually contain both fine-grained texture information and cross-region structural dependencies. Therefore, optimizing scanning strategies alone is insufficient to fully resolve the trade-off between preserving local details and capturing global structural patterns. Different from these approaches, this work does not redesign the 2D scanning mechanism. Instead, we focus on feature interaction within detection networks and propose CSSBlock, which integrates convolutional local priors with SS2D-based global state-space modeling to achieve local-global collaborative feature representation tailored for industrial defect detection scenarios.

### 2.4. Mixture of experts

As the scale of deep models continues to expand, network capacity has gradually become a critical bottleneck limiting performance. MoE, as a representative conditional computation mechanism, dynamically activates a small subset of expert subnetworks for different input samples, thereby substantially increasing model capacity without linearly increasing computational cost. Early studies introduced Sparsely-Gated MoE, demonstrating that this mechanism enables orders-of-magnitude expansion in model capacity for tasks such as language modeling and machine translation, while maintaining computational efficiency and significantly improving performance [54]. This work established both the theoretical and engineering foundations of MoE in large-scale deep models.

In recent years, MoE has been extended to broader modeling scenarios. In time-series modeling, researchers combined MoE with temporal- and frequency-domain feature modeling, leveraging expert specialization and routing mechanisms to adaptively model multi-pattern signals, thereby improving prediction performance on complex periodic and non-stationary sequences [55]. At the attention mechanism level, MoE has been employed to replace or restructure multi-head attention, enabling dynamic selection of effective attention subspaces conditioned on input content, thus enhancing inference efficiency and modeling flexibility without increasing parameter scale [56].

In vision and perception tasks, the advantages of MoE have gradually emerged. Some studies introduced MoE into event-camera-based object detection, designing experts with diverse perception mechanisms to achieve a favorable balance between accuracy and efficiency under challenging lighting and high-speed motion conditions [57]. In multi-task and multi-scenario perception systems such as autonomous driving, MoE enhances conditional adaptability of intermediate representations, allowing models to dynamically adjust computational paths according to environmental complexity and task requirements, thereby improving overall robustness and generalization [58]. In real-time object detection, YOLO-Master proposed the ES-MoE structure, which activates a small number of experts based on sample complexity through a lightweight routing network, effectively reducing redundant computation in simple scenarios while significantly improving detection performance in complex scenes [59].

Despite these advances highlighting the potential of MoE in improving model capacity and computational efficiency, its application to high-resolution industrial vision and structure-sensitive defect detection tasks remains limited. First, most existing MoE designs target natural scenes or general perception tasks, where expert specialization primarily focuses on semantic categories or scene complexity, providing insufficient modeling of fine-grained semantic variations caused by differences in material properties, manufacturing processes, and texture structures in industrial defects. Second, MoE modules are often directly embedded into backbones or detection heads as uniform dynamic computation units, without distinguishing the distinct modeling requirements of high-level semantic stages and low-level structural feature stages, which may lead to unstable routing or expert collapse. Finally, in real-time detection scenarios, introducing MoE mechanisms under strict inference budget constraints remains a key challenge for practical deployment.

Motivated by these observations, this paper further explores the integration of ES-MoE into a steel surface defect detection framework. By performing conditional expert modeling on high-level semantic features, the proposed approach enhances feature diversity and discriminability without significantly increasing computational overhead, thereby better adapting to industrial inspection scenarios characterized by large appearance variations and high inter-class similarity among defects.

## 3. Method

### 3.1. Network overview

**Fig 3:**
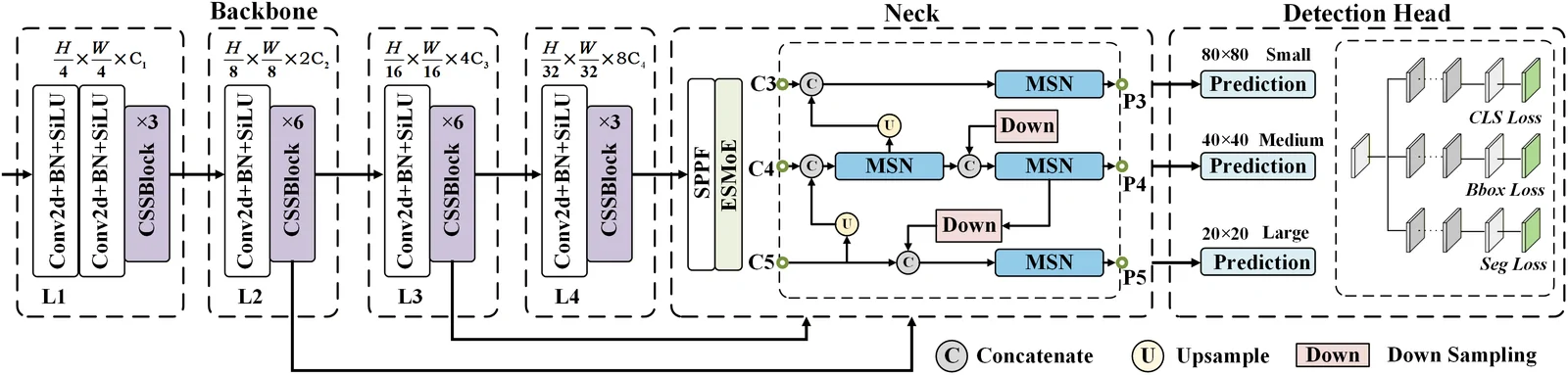
The overall architecture of the proposed EMamba.

As illustrated in Fig 3, the proposed EMamba is designed for high-resolution steel surface defect detection. The overall framework consists of three stages: feature extraction, cross-scale feature fusion, and target prediction. Each stage is specifically tailored to address key challenges in industrial defect inspection, including structural continuity, scale diversity, and discriminative precision.

In the feature extraction stage, the input steel surface image is first fed into a backbone network composed of CSSBlock and ES-MoE. By introducing a two-dimensional state space modeling mechanism, CSSBlock enables the network to capture long-range spatial dependencies on top of local convolutional priors, making it particularly suitable for modeling structural defects such as elongated cracks and continuous scratches. Meanwhile, ES-MoE is incorporated at the highest semantic stage of the backbone, where a sparse expert routing mechanism performs conditional feature transformation, enhancing the adaptive representation of complex defect patterns at the semantic level. The synergy between CSSBlock and ES-MoE equips the backbone with both strong local perception and global modeling capabilities without significantly increasing computational overhead, thereby providing high-quality features for subsequent multi-scale fusion.

To address the significant variations in scale, morphology, and semantic distribution of steel surface defects, MSN is introduced in the feature fusion stage as the core feature interaction module. MSN achieves an effective balance between high-order nonlinear interaction capability and computational efficiency. This hybrid design avoids redundant computation caused by uniformly enhancing all branches, while enabling stronger feature reorganization and selective enhancement at semantically critical stages. Through bidirectional top-down and bottom-up fusion pathways, MSN performs multi-scale semantic alignment and information compensation while maintaining structural stability, ultimately producing fused feature representations within a unified semantic space.

In the prediction stage, the fused multi-scale features are fed into a decoupled detection head for defect localization and classification. The detection head adopts independent regression and classification branches, focusing separately on geometric boundary modeling and defect category discrimination, thereby mitigating optimization conflicts caused by task coupling. This decoupled design significantly improves localization accuracy and classification stability in scenarios characterized by a high proportion of small defects and ambiguous boundaries, further enhancing overall detection performance.

### 3.2. Convolutional state space block

**Fig 4:**
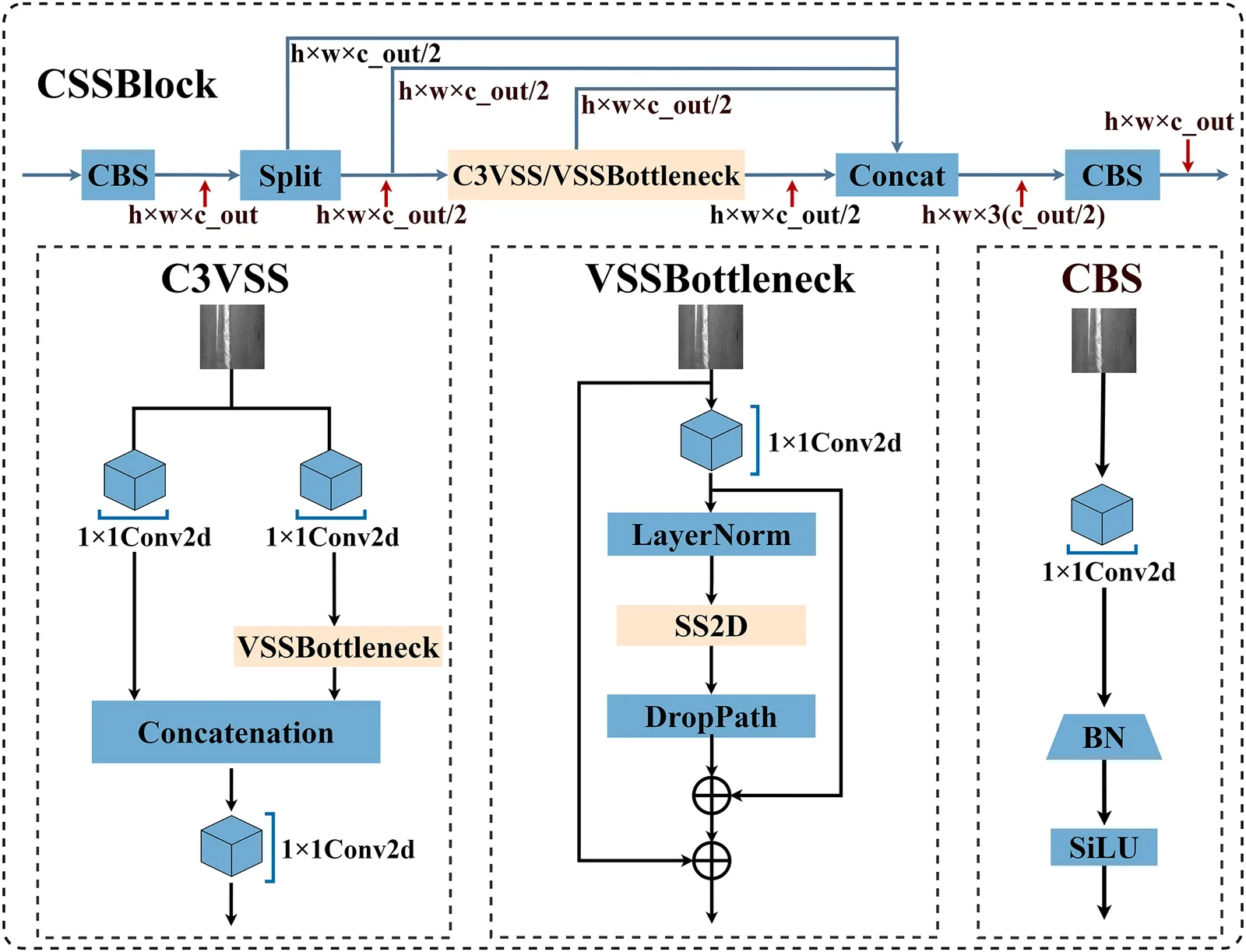
The overall architecture of the proposed CSSBlock.

As illustrated in Fig 4, we propose CSSBlock to simultaneously model local texture details and long-range spatial dependencies in steel surface defects. Different from simply inserting the VMamba module into the CSP structure or directly replacing convolution operations with SS2D, CSSBlock redesigns the internal feature interaction strategy of the CSP architecture by establishing a complementary relationship between local convolutional representations and state-space-based global modeling, thereby achieving more effective defect feature representation.

Existing CSP-based modules (e.g., C2f and C3k) in object detection backbones mainly rely on stacked convolutional operations for local feature extraction, and their modeling capability is constrained by the limited receptive field of convolution kernels. Although increasing network depth can gradually enlarge the effective receptive field, relying solely on local convolution is insufficient to fully capture cross-region spatial correlations for steel surface defects, such as elongated cracks, continuous scratches, and structurally consistent defects with directional patterns. On the other hand, the state-space model in VMamba can effectively establish long-range dependencies. However, directly introducing it as an independent module into the CSP structure ignores the complementary requirements between local texture details and global structural information in industrial defect detection.

Based on these observations, CSSBlock does not simply replace the convolutional units in CSP. Instead, it introduces a two-dimensional state-space modeling mechanism while preserving the local modeling capability of convolutional branches, thereby enhancing high-level semantic feature interactions. Specifically, the convolutional branch is responsible for extracting edge information, texture patterns, and fine-grained local variations within defect regions, while the SS2D-based state-space branch captures long-range spatial dependencies and overall structural patterns. Through the collaborative modeling of local and global features, CSSBlock simultaneously maintains the sensitivity of convolutional networks to detailed information and the capability of state-space models to represent complex structural relationships.

Furthermore, compared with directly applying the VMamba Block within the CSP structure, CSSBlock introduces additional structural optimization tailored to steel surface defect detection scenarios. Steel defects usually exhibit significant scale variations, blurred boundaries, and weak texture differences, making it difficult for either purely local modeling or purely global modeling to achieve optimal representations. Therefore, CSSBlock effectively integrates convolutional priors with dynamic state-space modeling capabilities, achieving efficient defect-oriented feature enhancement while maintaining linear computational complexity.

Given an input feature map $X\in {\mathbb{R}}^{B\times C\times H\times W}$, CSSBlock adopts a standard CSP structure. First, a 1 × 1 convolution projects the input features to an intermediate dimension and splits them along the channel dimension into a residual branch and a transformation branch. The residual branch serves as a shortcut path to preserve the original local information, while the transformation branch feeds the features into several VSS-based bottleneck modules for deep feature modeling. Finally, the outputs of the two branches are concatenated along the channel dimension and fused through a 1 × 1 convolution to produce the final output of the module.


$$\begin{array}{c} Y={𝒞}_{\text{merge}}\left(X_{\text{1}}\;\|\;{ℱ}_{\text{VSS}}\left(X_{\text{2}}\right)\right) \end{array}$$ (1)

Here, ${ℱ}_{\text{VSS}}(\cdot )$ denotes the feature transformation function composed of multiple VSS-Bottleneck modules. This design not only facilitates stable gradient propagation and efficient parameter utilization, but also endows the network with enhanced contextual modeling capability.

Instead of directly replacing convolution with SS2D, CSSBlock reformulates the feature transformation pathway by integrating convolutional local representation and SS2D-based state-space modeling within the CSP bottleneck. Specifically, given the intermediate feature U, the VSS-Bottleneck is formulated as:


$$\begin{array}{c} Z=U+{𝒮}_{\text{2D}}\left(U\right) \end{array}$$ (2)

Here, ${𝒮}_{\text{2D}}(\cdot )$ denotes the two-dimensional selective state space operator. This residual formulation not only stabilizes the training process, but also enables the state space modeling component to focus on capturing global dependencies without disrupting the original local feature distribution.

Unlike self-attention mechanisms, the computational complexity of the VSS-Bottleneck scales linearly with respect to the number of spatial positions. As a result, CSSBlock maintains favorable computational efficiency even under high-resolution inputs. Moreover, the module strictly follows the input–output interface design of C2f/C3k, allowing seamless replacement of existing CSP modules and ensuring strong engineering compatibility.

**Fig 5:**
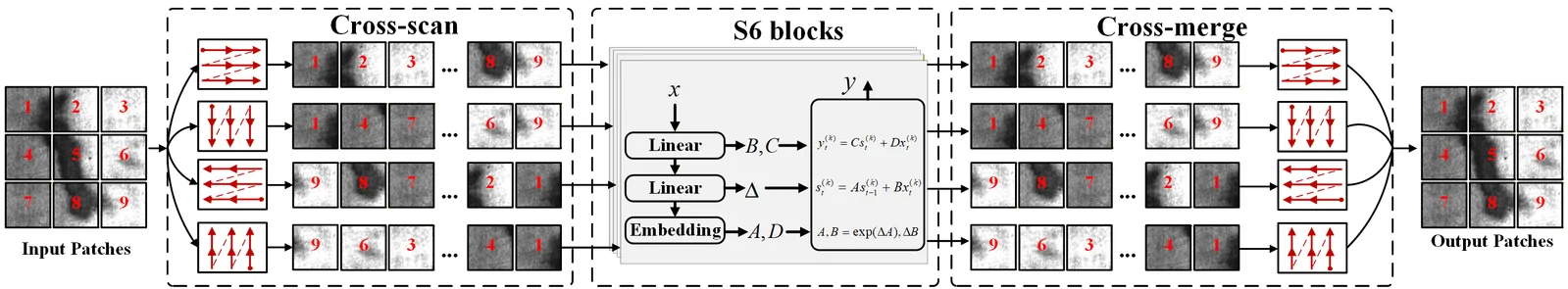
Working mechanism of SS2D.


As illustrated in Fig 5, to fully capture the directional characteristics of spatial structures, SS2D adopts a four-directional scanning mechanism, propagating states along the horizontal, vertical, and their corresponding reverse directions. For the *k*-th scanning direction, the state update process can be formulated as:

$$\begin{array}{c} {𝐬}_{t}^{\left(k\right)}=𝐀\text{\textbackslash{}hspace\{0.17em}\}{𝐬}_{t-1}^{\left(k\right)}+𝐁\text{\textbackslash{}hspace\{0.17em}\}{𝐱}_{t}^{\left(k\right)}\text{\textbackslash{}hspace\{0.33em}\}{𝐲}_{t}^{\left(k\right)}=𝐂\text{\textbackslash{}hspace\{0.17em}\}{𝐬}_{t}^{\left(k\right)}+𝐃\text{\textbackslash{}hspace\{0.17em}\}{𝐱}_{t}^{\left(k\right)} \end{array}$$ (3)

Here, ${𝐬}_{t}^{\left(k\right)}$ denotes the hidden state, and 𝐀\hspace{0.17em},𝐁\hspace{0.17em},𝐂,𝐃 represents the learnable state space parameters. The output features from the four scanning directions are subsequently fused to obtain the final response:


$$\begin{array}{c} 𝐘=\sum_{k=1}^{\text{4}}{𝐲}^{\left(k\right)} \end{array}$$ (4)

This multi-directional modeling strategy is particularly well-suited for target patterns in steel surface defects that exhibit strong directional continuity and long-range structural dependencies.

Overall, the innovation of CSSBlock does not lie in introducing the SS2D state-space model for the first time, but rather in proposing a convolutional–state-space collaborative modeling strategy tailored for industrial defect detection. Compared with a simple CSP-VMamba combination, CSSBlock reorganizes the information flow between local feature extraction and global dependency modeling, achieving a better balance among computational efficiency, fine-grained defect perception capability, and complex structural representation ability. This design provides an effective foundation for further improving steel surface defect detection performance.

### 3.3. Efficient sparse mixture-of-experts block

**Fig 6:**
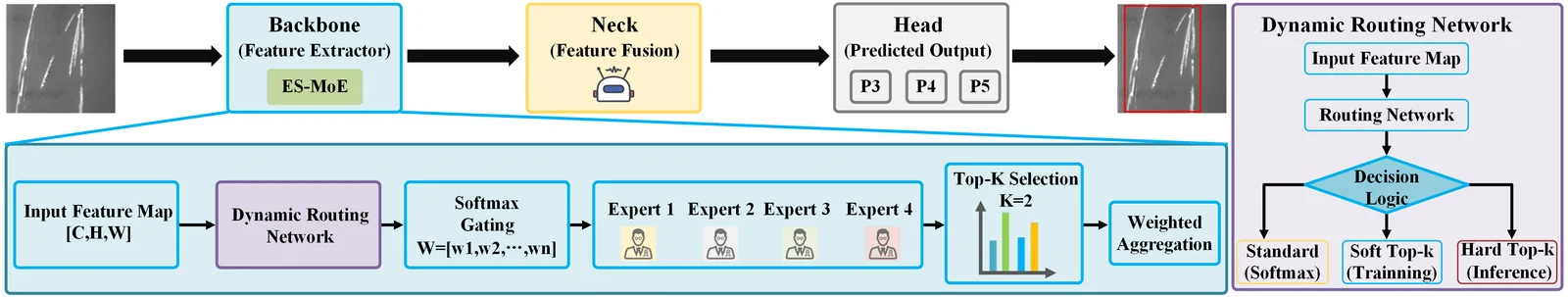
Working mechanism of ES-MoE.

As illustrated in Fig 6, ES-MoE is integrated into the highest semantic stage of the backbone network to enhance high-level feature modeling capability without introducing excessive computational overhead. It should be emphasized that ES-MoE does not directly adopt the Mixture-of-Experts mechanism from YOLO-Master. Instead, it redesigns the expert specialization strategy and feature routing mechanism according to the characteristics of steel surface defect detection.

In industrial surface defect detection, high-level backbone features need to simultaneously satisfy the following requirements: (1) strong semantic discrimination capability to distinguish different defect categories with highly similar appearances; (2) conditional feature modeling capability to adapt to variations among different defect patterns; and (3) low computational complexity to meet practical industrial deployment requirements. However, existing mainstream networks based on convolution or attention mechanisms generally adopt static parameter-sharing strategies, where all input samples are processed through the same feature transformation process. Such fixed representation mechanisms are insufficient to fully adapt to the variations in defect morphology, texture patterns, and structural complexity commonly observed in steel surface defects.

Although ES-MoE is inspired by the Mixture-of-Experts concept in YOLO-Master, it is not a direct reuse of the original MoE module. YOLO-Master mainly targets general object detection tasks and improves detection performance by enabling different experts to learn diverse object-level representations. In contrast, steel surface defect detection exhibits distinct task-specific characteristics, including weak visual differences among defect categories, significant intra-class variations, irregular defect structures, and prominent fine-grained texture changes. Therefore, directly applying a general-purpose MoE mechanism cannot fully satisfy the feature modeling requirements of industrial defect detection.

Given the high-level output feature map $X\in {\mathbb{R}}^{B\times C\times H\times W}$ from the backbone network, ES-MoE consists of three core components: 1) Gating router, which generates expert selection weights conditioned on the input features; 2) Expert subnetworks, which perform diverse feature transformations; 3) Sparse aggregation mechanism, which fuses only the outputs of a small subset of activated experts through weighted combination. The overall forward computation can be formulated as:


$$\begin{array}{c} Y=\sum_{i\in 𝒮\left(X\right)}g_{i}\left(X\right)\cdot E_{i}\left(X\right) \end{array}$$ (5)

Here, $E_{i}(\cdot )$ denotes the *i*-th expert, $g_{i}(\cdot )$ represents the corresponding gating weight, and 𝒮(X) denotes the sparsely selected set of experts determined by the router.

ES-MoE adopts a Top-k sparse routing strategy. The router first generates an expert response vector:


$$\begin{array}{c} 𝐳=ℛ\left(𝐗\right),\text{\textbackslash{}hspace\{0.33em}\;\;\;\}𝐳\in {\mathbb{R}}^{N} \end{array}$$ (6)

Where, N denotes the total number of experts. Only the top-k experts with the highest response values are selected to participate in the computation:


$$\begin{array}{c} 𝒮\left(𝐗\right)=\text{TopK}\left(𝐳,K\right) \end{array}$$ (7)

Subsequently, the responses of the selected experts are normalized to obtain the final gating weights:


$$\begin{array}{c} g_{i}\left(𝐗\right)=\frac{exp\left(z_{i}\right)}{\sum_{j\in 𝒮\left(𝐗\right)}exp\left(z_{j}\right)},\text{\textbackslash{}hspace\{0.33em}\;\;\;\}i\in 𝒮\left(𝐗\right) \end{array}$$ (8)

This design allows ES-MoE to preserve representational diversity while constraining computational complexity to scale proportionally with K, making it suitable for efficiency-sensitive detection frameworks.

Compared with the original MoE design, the fundamental contribution of ES-MoE does not lie in proposing a new expert mechanism, but in redesigning the expert specialization strategy and conditional feature selection scheme for industrial defect detection scenarios. By activating only a subset of experts for feature computation, ES-MoE enhances the representation capability for diverse defect patterns while effectively controlling additional computational overhead. This enables EMamba to achieve more flexible high-level semantic modeling capability while maintaining deployment efficiency.

### 3.4. Mixed star network

Existing multi-branch convolutional networks typically adopt uniform stacking or full replacement strategies when introducing enhanced feature interaction modules, i.e., applying the enhancement modules directly to all network layers or branches. However, such designs often overlook the differences among various branches and hierarchical levels in terms of semantic abstraction, information density, and modeling requirements. On the one hand, low-level stages mainly focus on extracting local textures, edges, and fine-grained details, where excessive introduction of complex high-order interaction modules may lead to unnecessary computational redundancy. On the other hand, high-level semantic features exhibit stronger abstraction and contextual dependencies, making it difficult for conventional convolution operations alone to sufficiently model complex defect structures.

The Star Block enhances feature reorganization capability through multiplicative interaction and channel-wise modulation mechanisms, providing an effective solution for high-order semantic modeling. However, directly applying Star Block to all network layers cannot fully exploit its advantages and may introduce additional computational overhead. Therefore, the core idea of this work is not to simply adopt the Star Block, but to propose a Selective High-order Feature Enhancement Strategy for multi-branch defect detection networks.

Based on this observation, we propose MSN, which analyzes the semantic feature requirements of different branches and hierarchical levels, and selectively introduces Star Block into critical positions with high semantic abstraction demands while preserving lightweight convolutional modeling in other positions. This design enhances the representation capability of complex defect structures while avoiding the computational redundancy caused by full-network enhancement, achieving a dynamic balance between detection performance and computational efficiency.

**Fig 7:**
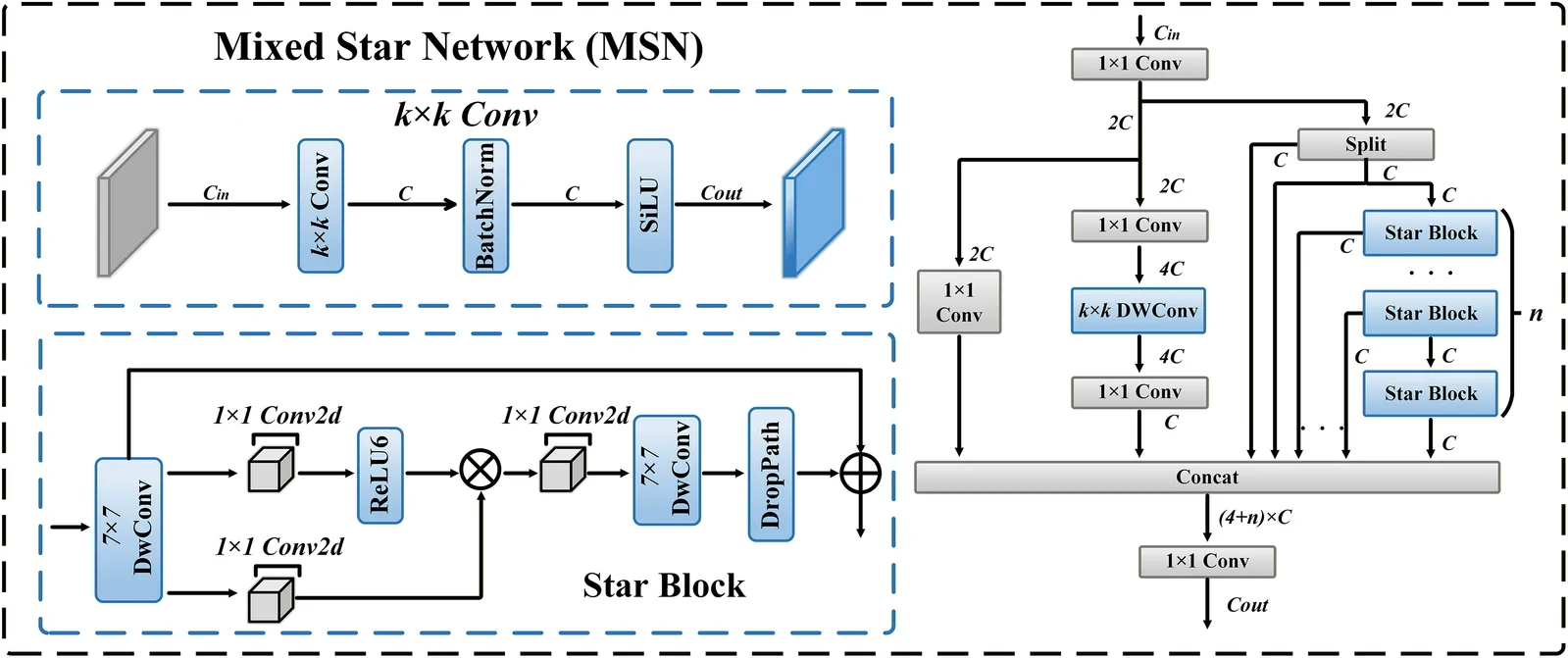
Structure of the proposed MSN.


As illustrated in Fig 7, MSN adopts a hybrid architecture consisting of channel mapping, multi-branch generation, and feature concatenation-fusion. Formally, let ${ℱ}_{l}(\cdot )$ denote the transformation function of the *l*-th module; then, the transformation in MSN can be expressed as:

$$\begin{array}{c} {ℱ}_{l}(\cdot )=\left\{ \begin{array}{cc} @ll@{ℱ}_{\text{Star}}(\cdot ), & l\in {ℒ}_{\text{Star}},\\ {ℱ}_{\text{Bottleneck}}(\cdot ), & \text{otherwise}. \end{array}\right. \end{array}$$ (9)

Here, ${ℒ}_{\text{Star}}$ denotes the set of selected stages or branches where the Star Block is inserted. This hybrid design enables the network to maintain overall structural consistency while acquiring stronger nonlinear modeling capability at semantically critical positions.

Compared with the original Star Block, the primary difference of MSN is that it is not directly embedded into the network as an independent general-purpose feature enhancement unit. Instead, MSN further considers the functional differences among different feature paths within multi-branch detection architectures. The original Star Block mainly focuses on high-order nonlinear interactions within a single feature stream and enhances feature representation by strengthening channel-wise relationships. In contrast, MSN analyzes the information transmission roles of different branches from the perspective of network architecture and selects appropriate positions for high-order modeling according to the semantic hierarchy of features.

Specifically, considering the characteristics of steel surface defect detection, including multi-scale distributions, weakly salient textures, and irregular structures, MSN treats Star Block as a high-order semantic enhancement unit rather than simply replacing conventional convolutional modules. In low-level feature branches, MSN retains the original convolution operations to fully exploit the advantages of convolutional networks in modeling local textures, edge details, and spatial continuity. In high-level semantic branches, Star Block is introduced to enhance feature reorganization capability, enabling the network to further capture long-range dependencies and complex structural information within defect regions. Through this hierarchical selection mechanism, MSN avoids the computational waste caused by introducing high-order modules throughout the entire network while improving the representation capability of critical semantic regions.

Furthermore, MSN does not merely focus on feature enhancement, but establishes a new balance among representation capability, computational complexity, and deployment efficiency. Compared with directly stacking Star Block modules, MSN controls the application scope of high-order interaction modules according to task requirements, allowing computational resources to be concentrated on feature levels that have a greater impact on detection performance. Therefore, while maintaining lightweight characteristics, MSN effectively improves the model’s perception capability for fine-grained defect patterns and complex structural variations.

In summary, the novelty of MSN lies in transforming Star Block from a general-purpose enhancement operator into a selectively deployed high-order semantic modeling component within multi-branch detection architectures. This paradigm goes beyond the conventional strategy of uniformly applying enhancement modules and achieves effective integration of existing high-order feature interaction mechanisms with industrial defect detection tasks through semantic hierarchy awareness and branch selection mechanisms. It provides a new perspective for designing high-performance and efficient detection networks in complex scenarios.

## 4. Experiments and analysis

### 4.1. Dataset description

To comprehensively evaluate the effectiveness and generalization capability of the proposed method across diverse steel surface defect scenarios, experiments are conducted on three representative public datasets: NEU-DET, GC10-DET, and Severstal Steel Defect Dataset. These datasets exhibit substantial differences in defect categories, scale distributions, image resolutions, and background complexity, collectively reflecting the diversity of real-world industrial inspection environments. Table 1 summarizes the defect categories and sample quantities of each dataset.

**Table 1:** Details of NEU-DET, GC10-DET and severstal datasets.

Dataset	Class type	Total images	Images for train	Images for val	Images for test
NEU-DET	6	1800	1440	180	180
GC10-DET	10	2291	1832	229	230
Severstal	4	6666	5332	667	667

1) NEU-DET is a classical steel surface defect dataset released by Northeastern University and is widely used for benchmarking defect detection algorithms. It contains six typical defect categories: Crazing (Cr), Patches (Pa), Inclusion (In), Pitted Surface (PS), Rolled-in Scale (RS), and Scratches (Sc). Each category includes 300 grayscale images with a resolution of 200 × 200. Although the defect patterns are relatively clear, the scale variation is significant, and certain categories exhibit high visual similarity. Consequently, the dataset imposes strict requirements on fine-grained discrimination and small-object detection capability. The dataset is available at https://www.kaggle.com/datasets/mazilishanglx/neu-det.
2) GC10-DET is a larger and more challenging steel surface defect dataset comprising 10 defect categories and a total of 2,291 images. It presents greater diversity in defect morphology, size distribution, and background texture. Some defects exhibit irregular boundaries, elongated structures, or low-contrast characteristics. Compared with NEU-DET, GC10-DET more closely resembles real industrial production environments, posing higher challenges in multi-scale modeling, complex background suppression, and inter-class discrimination. The dataset is available at https://www.kaggle.com/datasets/mazilishanglx/gc10-det
3) The Severstal Steel Defect Dataset is released by industry and collected from real steel production lines, featuring higher image resolution and stronger engineering relevance. It contains four categories of steel surface defects, primarily high-resolution grayscale images. The defects often exhibit uneven scales, elongated shapes, and ambiguous boundaries. Compared with academic datasets, Severstal more closely reflects real-world industrial conditions in terms of imaging noise, illumination variations, and class imbalance, thereby imposing stricter requirements on model robustness, generalization, and high-resolution modeling capability. The dataset is available at https://www.kaggle.com/datasets/mazilishanglx/severstal.

### 4.2. Training strategies and implementation details

To ensure reproducibility and fairness, all experiments are conducted under a unified hardware and software environment. Specifically, the experimental platform is equipped with an Intel i9-13900K 3.00 GHz CPU and an NVIDIA GeForce RTX 3090 GPU (24 GB memory), running on Windows 10. Model training and inference are implemented using Python 3.8 with the PyTorch 2.2.2 deep learning framework, accelerated by CUDA 11.8.

In terms of training strategy, the model is optimized in an end-to-end supervised manner. The initial learning rate is set to 0.001, and a 3-epoch warm-up phase is introduced at the beginning of training to alleviate instability during early optimization. Subsequently, a cosine annealing learning rate schedule is adopted to gradually decrease the learning rate in later stages, enabling finer parameter updates and improved convergence quality.

For optimization, Adam is employed as the parameter update algorithm. Compared with conventional SGD, Adam adaptively adjusts learning rates for different parameters and mitigates overfitting through decoupled weight decay, making it particularly suitable for steel surface defect detection tasks characterized by complex backgrounds and significant scale variations. The momentum coefficient is set to 0.937, and the weight decay coefficient is set to 5 × 10^-4^, achieving a favorable balance between convergence speed and training stability. The model is trained for 200 epochs with a batch size of 8, considering both GPU memory constraints and training efficiency.

Regarding data preprocessing and inference settings, all input images are resized to 640 × 640 during training. The same input resolution is maintained during inference to avoid performance shifts caused by scale inconsistency. During detection, the confidence threshold is set to 0.25, and the IoU threshold for non-maximum suppression (NMS) is set to 0.45, striking a reasonable balance between recall and false positives.

The above training strategies and implementation details are kept consistent across different datasets and experimental configurations, ensuring fairness in comparisons and reliability of the reported results.

### 4.3. Analysis of optimization strategy

The optimizer plays an important role in training deep detection networks, especially for models with complex feature interaction mechanisms. To investigate the influence of different optimization strategies on EMamba, we compare three commonly used optimizers, including SGD, AdamW, and Adam, under the same experimental settings. As shown in Table 2, SGD achieves a reasonable detection performance with 78.9% mAP50, demonstrating that it can effectively optimize the proposed architecture. However, AdamW and Adam obtain better results by introducing adaptive learning rate adjustment. Among them, Adam achieves the best performance, reaching 80.4% mAP50 and 48.6% mAP50-95, outperforming SGD by 1.5 and 1.4 percentage points, respectively.

**Table 2:** Ablation study of different optimizers on the NEU-DET dataset.

Optimizer	P↑	R↑	mAP_50_↑	mAP_50-95_↑
SGD	0.716	0.704	78.9	47.2
AdamW	0.731	0.721	79.8	48.0
Adam	0.742	0.731	80.4	48.6

The improvement can be attributed to the adaptive optimization mechanism of Adam, which provides more flexible parameter updates for the heterogeneous feature representations introduced by CSSBlock, MSN, and ES-MoE. Therefore, Adam is selected as the default optimizer for EMamba to achieve more stable convergence and better detection performance.

**Statistical Analysis.** To assess the robustness and statistical reliability of the experimental results, YOLO11n and EMamba were independently trained five times using different random seeds while keeping the training configuration unchanged. The detection performance was summarized using the mean and standard deviation (SD) across the five independent runs. To evaluate whether the performance differences between YOLO11n and EMamba were statistically significant, a two-sided Welch’s *t*-test was conducted for the main detection metrics. A *p*-value of less than 0.05 was considered statistically significant.

### 4.4. Evaluation metrics

To comprehensively evaluate the performance of the proposed object detection model, the following metrics are adopted [60]:

1) Precision (P) measures the proportion of correctly predicted positive samples among all positive predictions. It reflects the model’s ability to reduce false positives and is defined as:

$$\begin{array}{c} Precision=\frac{TP}{TP+FP} \end{array}$$ (10)

Where, TP refers to targets accurately detected by the model, while FP indicates background areas wrongly predicted as objects.

2) Recall (R) evaluates the proportion of correctly predicted positive samples among all actual positive samples, indicating the model’s sensitivity to detecting objects:

$$\begin{array}{c} Recall=\frac{TP}{TP+FN} \end{array}$$ (11)

Where, FN represents the objects missed by the detector, which are mistakenly classified as background.

3) Average Precision (AP) quantifies the area under the Precision-Recall (P-R) curve for a specific class and IoU threshold. It provides a balanced evaluation of detection accuracy:

$$\begin{array}{c} AP_{n}=\int_{\text{0}}^{\text{1}}P\left(x\right)dx \end{array}$$ (12)

Where, n denotes the number of categories, and P(x) represents the Precision-Recall curve for category AP. The area under this curve, obtained via integration, corresponds to the AP value for that category.

4) Mean Average Precision (mAP) is the average AP across all classes and multiple IoU thresholds:

$$\begin{array}{c} mAP=\frac{\text{1}}{N}\sum_{i-1}^{N}AP_{n} \end{array}$$ (13)

Where N denotes the total number of categories, and $AP_{n}$ represents the Average Precision (AP) value for category n.

5) FPS: To evaluate the real-time performance of the detector, FPS is used as a key metric. FPS indicates the number of images the detector can process per second, providing an assessment of its inference speed. A higher FPS value signifies improved real-time performance of the detector.

$$\begin{array}{c} FPS=\frac{\text{1}}{T_{m}} \end{array}$$ (14)

Where, $T_{m}$ denotes the time taken by the detector to process a single defect image.

6) Parameters and Computational Complexity: Params and GFLOPs are important metrics for evaluating the lightweight nature of a model. Params refers to the number of parameters in the model, the larger the parameter count, the heavier the model weights. GFLOPs indicates the number of floating-point operations required by the model, higher GFLOPs imply greater computational cost and slower inference speed. Taking a convolution operation with a kernel size of as an example, the definitions of Params and GFLOPs are as follows:

$$\begin{array}{c} Params=C_{out}\times (k_{i\times i}^{\text{2}}\times C_{in}+1) \end{array}$$ (15)

$$\begin{array}{c} GFLOPs=\frac{2\times C_{in}\times k_{i\times i}^{\text{2}}\times C_{out}\times W_{i}\times H_{i}}{10^{\text{9}}} \end{array}$$ (16)

where $C_{in}$ denotes the number of input channels, $k_{i\times i}$ represents a convolution with kernel size i×i, $C_{out}$ denotes the number of output channels, $W_{i}$ and $H_{i}$ represent the width i and height i of the feature map, respectively.

### 4.5. Results on NEU-DET dataset

To validate the effectiveness of the proposed EMamba for steel surface defect detection, we conduct comprehensive comparisons on the NEU-DET dataset against a broad range of representative object detection methods. The evaluated detectors include lightweight YOLO-series models (e.g., YOLOv5–YOLOv13 and Hyper-YOLO), two-stage detectors such as Faster R-CNN, single-stage detectors including SSD, EfficientDet, and FCOS, as well as representative steel defect detection approaches, including MSC-DNet, CS-YOLO, CABF-FCOS, DFFNet, LFF-YOLO, DCC-CenterNet, RSTD-YOLOv7, and TD-Net. Furthermore, to investigate the effectiveness of the proposed hybrid modeling paradigm compared with recent advanced architectures, we additionally introduce Mamba-based and Transformer-based detectors, including Mamba-YOLO-T, RT-DETR, and DINO, for further comparison.

As shown in Table 3, EMamba achieves the best overall detection performance on the NEU-DET dataset, obtaining 80.4% mAP50 and 48.6% mAP50-95, which demonstrates its advantages in both defect localization and accurate bounding box regression. Compared with other YOLO-based detectors, EMamba consistently improves detection accuracy while maintaining a lightweight architecture with only 3.68M parameters and 114.6 FPS inference speed. In addition, compared with recent Mamba-based and Transformer-based detectors, EMamba also exhibits competitive advantages. Specifically, EMamba outperforms Mamba-YOLO-T by 1.8 percentage points in mAP50 while using fewer parameters, demonstrating the effectiveness of the proposed hybrid modeling strategy. Compared with RT-DETR and DINO, EMamba achieves higher detection accuracy with substantially lower computational complexity, indicating a better balance between representation capability and deployment efficiency.

**Table 3:** Performance comparison of each method on NEU-DET datasets.

Method	P↑	R↑	mAP_50_↑	mAP_50-95_↑	AP_s_↑	AP_m_↑	AP_l_↑	Params↓	FPS↑
YOLOv5n	0.637	0.714	72.9	42.9	30.1	55.0	66.7	2.65	184.1
YOLOv6n	0.681	0.705	73.8	43.6	31.0	55.8	67.4	4.50	**226.4**
YOLOv8n	0.668	0.710	74.6	44.2	32.2	56.6	68.0	3.15	198.1
YOLOv8s	0.698	0.689	74.2	44.5	33.0	57.2	68.6	11.16	170.4
YOLOv9c	0.616	**0.741**	73.0	42.8	30.7	55.4	66.9	25.59	51.5
YOLOv10n	0.634	0.639	69.4	39.3	26.8	50.7	63.6	2.77	136.2
YOLOv10s	0.671	0.683	70.3	40.1	27.6	51.6	64.5	8.12	139.8
YOLOv11n	0.681 ± 0.004	0.671 ± 0.005	73.9 ± 0.004	43.4 ± 0.3	31.4 ± 0.4	56.1 ± 0.3	67.6 ± 0.3	2.58	135.3
YOLOv11s	0.717	0.677	75.1	44.9	33.7	57.7	69.0	9.43	134.1
YOLOv12n	0.701	0.694	74.4	44.1	32.8	56.9	68.3	2.55	151.4
YOLOv12s	0.686	0.680	74.4	44.3	33.1	57.1	68.5	9.23	147.0
YOLOv13n	0.605	0.611	63.1	32.6	19.1	45.1	58.6	**2.44**	101.3
Hyper-YOLO	0.712	0.676	73.0	42.7	31.0	55.3	66.8	3.95	112.1
Faster-rcnn-resnet50	0.242	0.572	38.6	20.4	9.8	27.9	39.5	136.79	18.6
SSD-resnet50	0.781	0.093	36.1	18.2	8.9	26.1	38.0	12.33	53.6
EfficientDet-d1	0.643	0.261	44.8	24.7	12.6	32.9	45.1	6.55	19.9
FCOS	**0.781**	0.551	70.7	41.1	29.5	54.0	65.9	32.13	35.6
MSC-DNet [61]	–	–	79.4	–	–	–	–	34.1	14.1
CS-YOLO [62]	–	–	78.2	–	–	–	–	24.4	87
CABF-FCOS [63]	–	–	76.7	–	–	–	–	56.3	18
DFFNet [11]	–	–	80.2	–	–	–	–	10.3	101
LFF-YOLO [64]	–	–	79.2	–	–	–	–	60.5	63.2
DCC-CenterNet [65]	–	–	79.4	–	–	–	–	32.8	71.3
RSTD-YOLOv7 [34]	0.751	0.715	79.3	–	–	–	–	16.5	41.3
TD-Net [39]	–	–	76.8	–	–	–	–	7.06	–
Mamba-YOLO-T [66]	0.724	0.716	78.6	46.8	36.1	58.9	69.7	5.66	155
RT-DETR [32]	0.713	0.702	77.8	46.1	34.8	58.0	68.9	8.33	93
DINO [31]	0.756	0.728	79.1	47.5	36.7	59.4	70.1	47.55	48
Emamba (ours)	0.742 ± 0.003	0.731 ± 0.004	**80.4** ± 0.2	**48.6** ± 0.2	**38.9** ± 0.3	**60.4** ± 0.2	**71.2** ± 0.3	3.68	114.6

**Fig 8:**
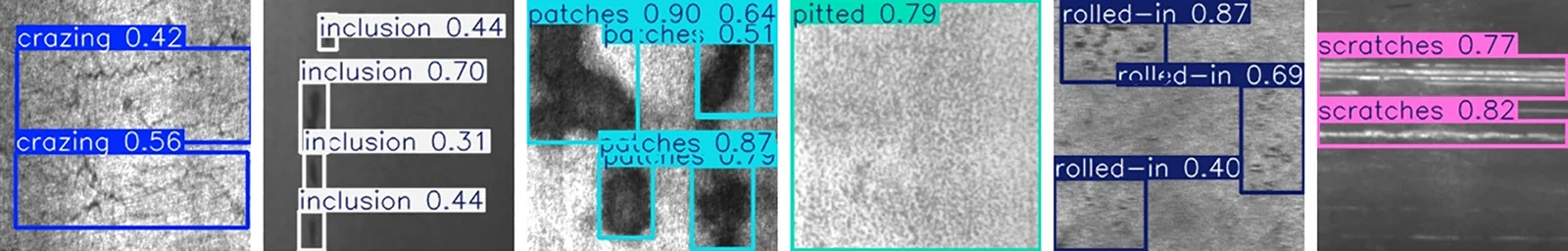
Detection results of EMamba for different defect categories on the NEU-DET dataset.

The superior performance of EMamba benefits from the complementary contributions of its three components. CSSBlock introduces state-space-based long-range dependency modeling while preserving local defect details, improving the perception of complex textures and structural patterns. MSN enhances hierarchical feature aggregation, enabling more effective representation of defects with different scales. ES-MoE further provides conditional feature modeling through expert routing, allowing the network to adaptively capture diverse defect characteristics. As a result, EMamba achieves competitive performance on small, medium, and large defect detection, with APs, APm, and APl reaching 38.9%, 60.4%, and 71.2%, respectively. These results verify the effectiveness of the proposed framework for robust steel surface defect detection. Fig 8 further provides qualitative detection results of EMamba on the NEU-DET dataset.

It is worth noting that some defect detection methods, such as MSC-DNet, DFFNet, and CS-YOLO, achieve comparable mAP50 values to EMamba. This is mainly because NEU-DET is a relatively mature benchmark with limited defect categories, where different advanced models may approach a similar performance saturation level. Therefore, the performance gap measured by mAP50 alone becomes relatively narrow.

Nevertheless, EMamba demonstrates clear advantages beyond the mAP50 metric. Compared with DFFNet, which achieves 80.2% mAP50, EMamba obtains a comparable but slightly higher detection accuracy while reducing the parameter number from 10.3M to 3.68M and improving inference speed from 101 FPS to 114.6 FPS. Compared with MSC-DNet, EMamba achieves a 1.0 percentage-point improvement in mAP50 with substantially fewer parameters and higher inference efficiency. These results indicate that EMamba provides a better balance between detection accuracy, model complexity, and practical deployment efficiency.

In addition, the improvement of EMamba mainly benefits from its ability to model long-range structural dependencies and conditional feature representations through CSSBlock and ES-MoE, which is particularly beneficial for complex defect patterns that require fine-grained structural perception.

Statistical analysis using a two-sided paired *t*-test showed that the performance improvements of EMamba over YOLOv11n were statistically significant (*p* < 0.05).

To evaluate the multi-defect detection capability of EMamba in complex real-world scenarios, we design a multi-object coexistence inference experiment. Specifically, images from the six defect categories of the NEU-DET are concatenated at equal resolution to construct a composite image, simulating a typical industrial scenario in which multiple defects appear simultaneously. Without any additional training or hyperparameter tuning, the trained EMamba model is directly applied to this composite image for inference.

**Fig 9:**
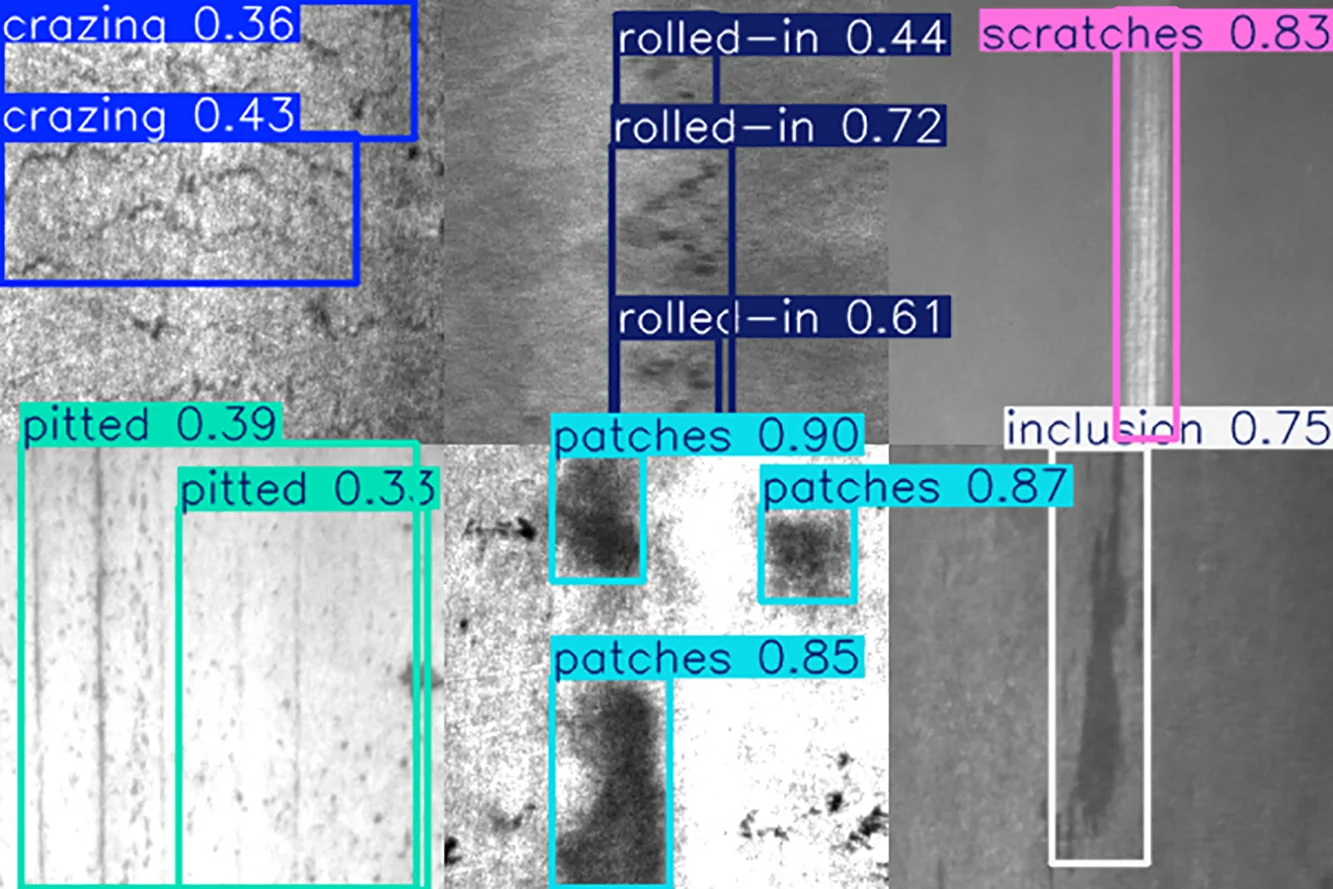
Inference results of EMamba on a composite image with multiple coexisting defects from the NEU-DET dataset.

As illustrated in Fig 9, the model accurately identifies and localizes all defect categories in the image. The predicted bounding boxes are precisely aligned with the targets, and the category predictions are correct, with no obvious missed detections or false positives. These results demonstrate that EMamba maintains high detection accuracy even under complex inputs where multiple defect types coexist and background interference is intensified, highlighting its strong generalization capability and robustness.

From a methodological perspective, CSSBlock enhances local feature discriminability, enabling accurate separation of adjacent defect edges and textures. The conditional expert mechanism of ES-MoE effectively adapts to different defect types and background variations, facilitating adaptive multi-object feature modeling. Meanwhile, MSN ensures comprehensive multi-scale feature fusion, providing precise localization across defect sizes ranging from small targets to large structural anomalies. The synergy among these components leads to superior multi-defect recognition performance in complex industrial environments.

Overall, this inference experiment not only validates the practicality of EMamba in multi-object coexistence scenarios but also further confirms the effectiveness of the proposed modules in enhancing feature discriminability and multi-object perception capability, offering strong support for deployment in real-world production lines.

**Fig 10:**
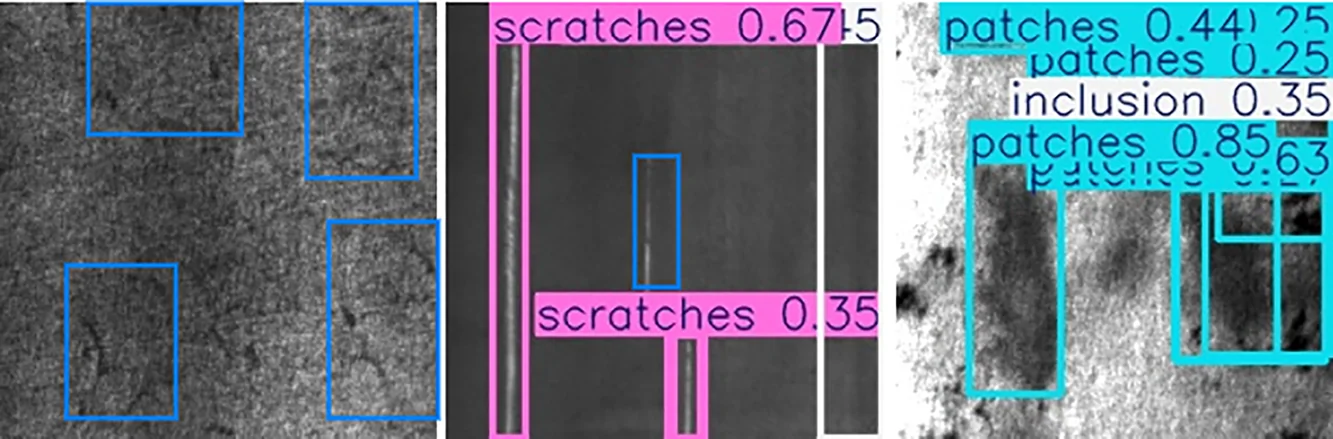
Representative failure cases of EMamba on the NEU-DET test set, including missed detections under low-light conditions, missed subtle scratch defects, and duplicate bounding boxes generated for the same defect region. Blue boxes denote the ground-truth annotations.

To comprehensively evaluate the robustness of EMamba in real-world industrial steel surface defect inspection, we further analyze representative failure cases on the NEU-DET test set. Although EMamba achieves stable and superior performance across most defect categories, certain limitations remain under a small number of challenging imaging conditions. As illustrated in Fig 10, the primary failure modes can be summarized into three categories.

First, under low-light imaging conditions, the overall contrast of steel surfaces is significantly reduced, and the intensity difference between defect regions and surrounding backgrounds becomes compressed. As a result, the discriminative responses of defect-related features are weakened, leading to occasional missed detections. Second, for extremely small scratch defects with subtle texture variations, their boundaries are highly similar to background patterns. The limited feature discrepancy makes it difficult for the detector to establish stable responses during localization, resulting in missed detections of weak defects. Finally, a small number of duplicate predictions can be observed in some cases, where multiple highly overlapped bounding boxes are generated for the same defect instance. This phenomenon mainly occurs in dense candidate regions with similar confidence scores, indicating that redundant prediction suppression can be further improved.

Further analysis shows that although CSSBlock effectively enhances fine-grained texture representation and improves the perception of small defects, extremely weak contrast conditions may still limit the reliability of feature responses. Meanwhile, the complex multi-scale characteristics of steel defects make it challenging for a fixed post-processing strategy to optimally handle different defect categories and spatial distributions. In the current implementation, standard NMS with a fixed IoU threshold is adopted to ensure fair comparison with existing detectors. However, this strategy may not fully adapt to the diverse morphological characteristics of industrial defects.

These failure cases provide several directions for future improvement, including enhancing feature contrast modeling under low-light conditions, strengthening fine-grained representation for subtle defects, and introducing more adaptive prediction suppression strategies. For practical deployment, category-aware NMS with dynamically adjusted IoU thresholds can be considered to better accommodate defects with different shapes and densities. In addition, Soft-NMS can be further explored to gradually reduce the confidence scores of overlapping predictions rather than directly removing them, thereby reducing duplicate detections while preserving valid predictions for complex defect patterns. Overall, although a small number of limitations remain under extreme conditions, EMamba maintains high detection accuracy and robustness in most industrial inspection scenarios, demonstrating its potential for practical deployment.

### 4.6. Results on GC10-DET dataset

To evaluate the generalization capability of EMamba for multi-class steel surface defect detection, we conduct comprehensive experiments on the GC10-DET dataset and compare it with a wide range of representative detectors. The evaluated methods include lightweight YOLO-series models (YOLOv3-tiny and YOLOv5–YOLOv13), optimized YOLO variants (YOLOv8-ghost and YOLOv8-world series), as well as recent Mamba-based and Transformer-based detectors, including Mamba-YOLO-T, RT-DETR, and DINO.

As shown in Table 4, EMamba achieves the best overall performance on GC10-DET, obtaining 64.9% mAP50 and 39.1% mAP50-95. Compared with YOLOv8-ghost, which achieves competitive performance with a lightweight architecture, EMamba improves mAP50 by 3.5 percentage points while maintaining a moderate computational cost. Furthermore, compared with Mamba-YOLO-T, EMamba increases mAP50 from 62.7% to 64.9%, demonstrating the advantage of the proposed hybrid modeling strategy over a single state-space modeling framework. Compared with Transformer-based detectors such as RT-DETR and DINO, EMamba achieves higher detection accuracy with fewer parameters and better inference efficiency, indicating a more favorable balance between representation capability and deployment requirements.

**Table 4:** Performance comparison of each method on GC10-det datasets.

Method	P↑	R↑	mAP_50_↑	mAP_50-95_↑	Params↓	FPS↑	GFLOPS↓
YOLOv3-tiny	0.505	0.571	53.2	30.4	12.17	**226.8**	19.1
YOLOv5n	0.548	0.471	51.4	28.6	2.65	184.1	7.8
YOLOv6n	0.543	0.513	51.2	28.9	4.50	226.4	13.1
YOLOv8n	0.513	0.527	53.2	30.8	3.15	198.1	**5.8**
YOLOv8-ghost	0.602	0.582	61.4	36.9	**1.86**	157.5	8.9
YOLOv8-world	0.587	0.548	57.4	33.7	4.20	124.6	39.6
YOLOv8-worldv2	0.504	**0.599**	55.9	33.1	3.69	139.5	19.5
YOLOv9c	0.512	0.496	51.8	29.0	25.59	51.5	104
YOLOv10n	0.660	0.514	56.7	34.2	2.77	136.2	8.7
YOLOv11n	**0.740** ± 0.003	0.545 ± 0.005	59.1 ± 0.5	36.0 ± 0.4	2.58	135.3	6.3
YOLOv12n	0.535	0.517	52.6	30.5	2.55	151.4	6.5
YOLOv13n	0.549	0.455	45.4	24.6	2.44	101.3	6.4
RSTD-YOLOv7	0.721	0.581	62.1	37.4	16.50	41.3	–
TD-Net	0.612	0.563	60.8	36.5	7.06	–	–
Mamba-YOLO-T	0.693	0.584	62.7	38.0	5.66	155	12.3
RT-DETR	0.676	0.571	61.5	37.1	8.33	93	16.1
DINO	0.714	0.588	63.2	38.3	47.55	48	–
Emamba (ours)	0.648 ± 0.005	0.596 ± 0.006	**64.9** ± 0.4	**39.1** **± 0.3**	3.68	112.9	7.7

The performance improvement of EMamba can be attributed to the complementary effects of its three components. CSSBlock enhances the modeling of local textures and long-range structural dependencies, enabling more discriminative representations for fine-grained defect patterns. MSN improves hierarchical feature aggregation, which facilitates the detection of defects with significant scale variations. ES-MoE introduces conditional expert routing to adaptively capture diverse defect characteristics under complex backgrounds and multi-category scenarios. These designs jointly improve detection accuracy while preserving computational efficiency.

**Fig 11:**
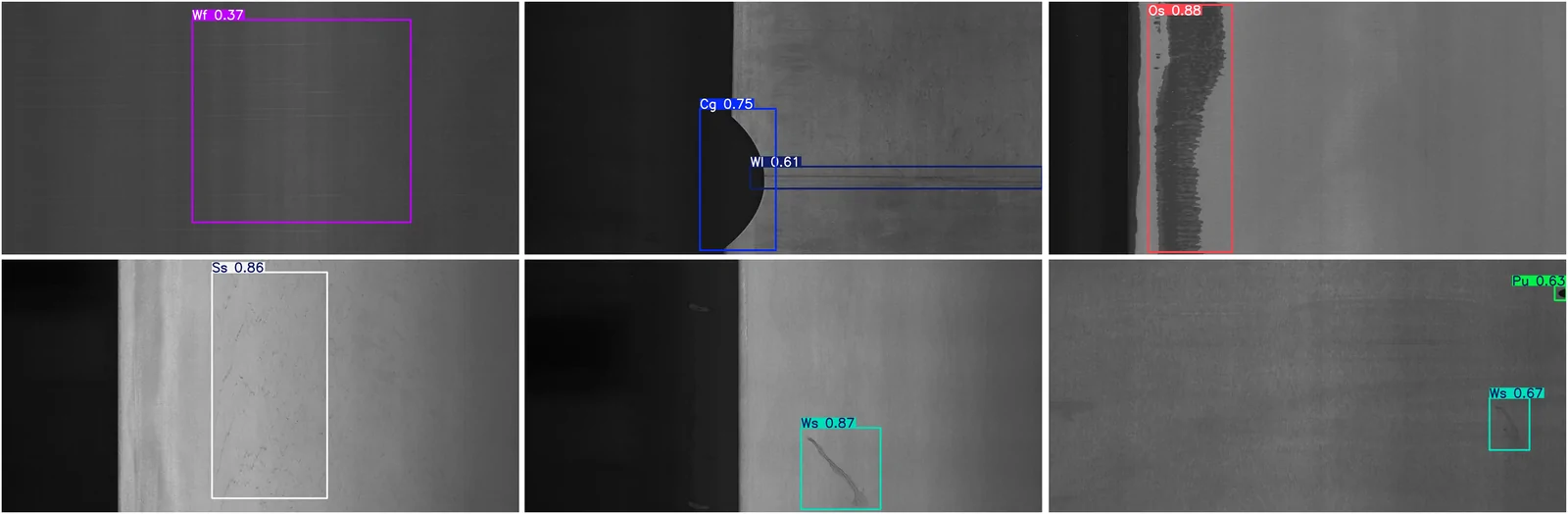
Detection results of EMamba for different defect categories on the GC10-DET dataset.

Overall, the results on GC10-DET demonstrate that EMamba provides robust detection capability for diverse steel surface defects and validates the effectiveness of the proposed hybrid modeling framework. Fig 11 further presents qualitative detection results of EMamba on the GC10-DET dataset.

### 4.7. Results on severstal dataset

To assess the practical applicability of EMamba in real-world industrial steel defect inspection, we conduct systematic evaluations on the Severstal Steel Defect Dataset. This dataset contains multiple defect categories, such as cracks, pores, and inclusions, with large variations in defect size and highly imbalanced distributions, providing a more challenging validation environment for detection models.

As reported in Table 5, EMamba achieves 52.1% mAP50 and 31.3% mAP50-95, outperforming all competing methods. It also maintains a favorable balance between Precision and Recall. Compared with Hyper-YOLO and YOLOv7-tiny, EMamba demonstrates clear improvements in recall for small defects and localization accuracy for large defects, indicating strong adaptability to multi-scale and multi-type defect scenarios. Moreover, while maintaining superior detection performance, EMamba preserves efficient inference speed, highlighting its potential for deployment in real industrial production lines.

**Table 5:** Performance comparison of each method on severstal datasets.

Methods	Params↓	GFLOPS↓	FPS↑	mAP_50_↑	mAP_50-95_↑	P↑	R↑
YOLOv5n	2.65	**4.1**	**208.2**	43.5	24.6	0.508	0.439
YOLOv7-tiny	6.03	13	206.1	50.2	29.8	0.538	0.495
YOLOv8n	3.15	8.1	188.4	46.4	26.3	0.522	0.441
GELAN-t	**1.87**	7.1	73.6	49.7	29.4	0.537	0.489
YOLOv10n	2.70	8.2	125.1	46.5	27.1	0.497	0.490
YOLOv11n	2.58	6.3	135.3	44.6 ± 0.4	25.5 ± 0.3	0.518 ± 0.006	0.457 ± 0.007
YOLOv12n	2.55	6.5	151.4	46.4	26.8	0.499	0.476
YOLOv13n	2.44	6.4	101.3	32.3	17.6	0.398	0.367
Hyper-YOLO	3.90	10.8	113.7	49.7	30.6	**0.550**	0.502
Emamba (ours)	3.68	7.7	114.2	**52.1** ± 0.3	**31.3** ± 0.3	0.534 ± 0.005	**0.516** **± 0.004**

**Fig 12:**
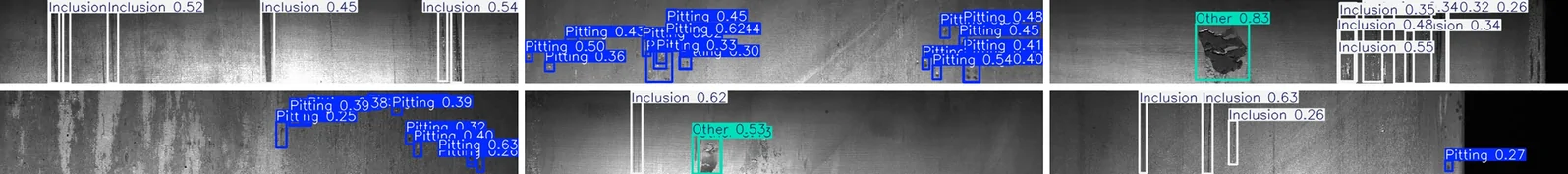
Detection results of EMamba for different defect categories on the Severstal dataset.

Overall, the results on the Severstal dataset further confirm the advantages of EMamba in handling diverse defect types, multi-scale variations, and complex surface textures. They also validate the effectiveness of the three core modules in enhancing detection robustness and generalization capability. Fig 12 presents qualitative detection results of EMamba on the Severstal dataset.

### 4.8. Domain-shift robustness evaluation

To evaluate the practical applicability of EMamba in real-world steel production environments, we conduct a systematic domain shift robustness analysis on the NEU-DET test set. Public datasets are typically carefully curated and exhibit relatively high image quality. In contrast, industrial steel inspection scenarios often involve low contrast, strong specular reflections, high noise levels, motion blur, non-uniform illumination, complex rolling textures, and partial occlusions, all of which can induce significant domain shifts.

**Fig 13:**
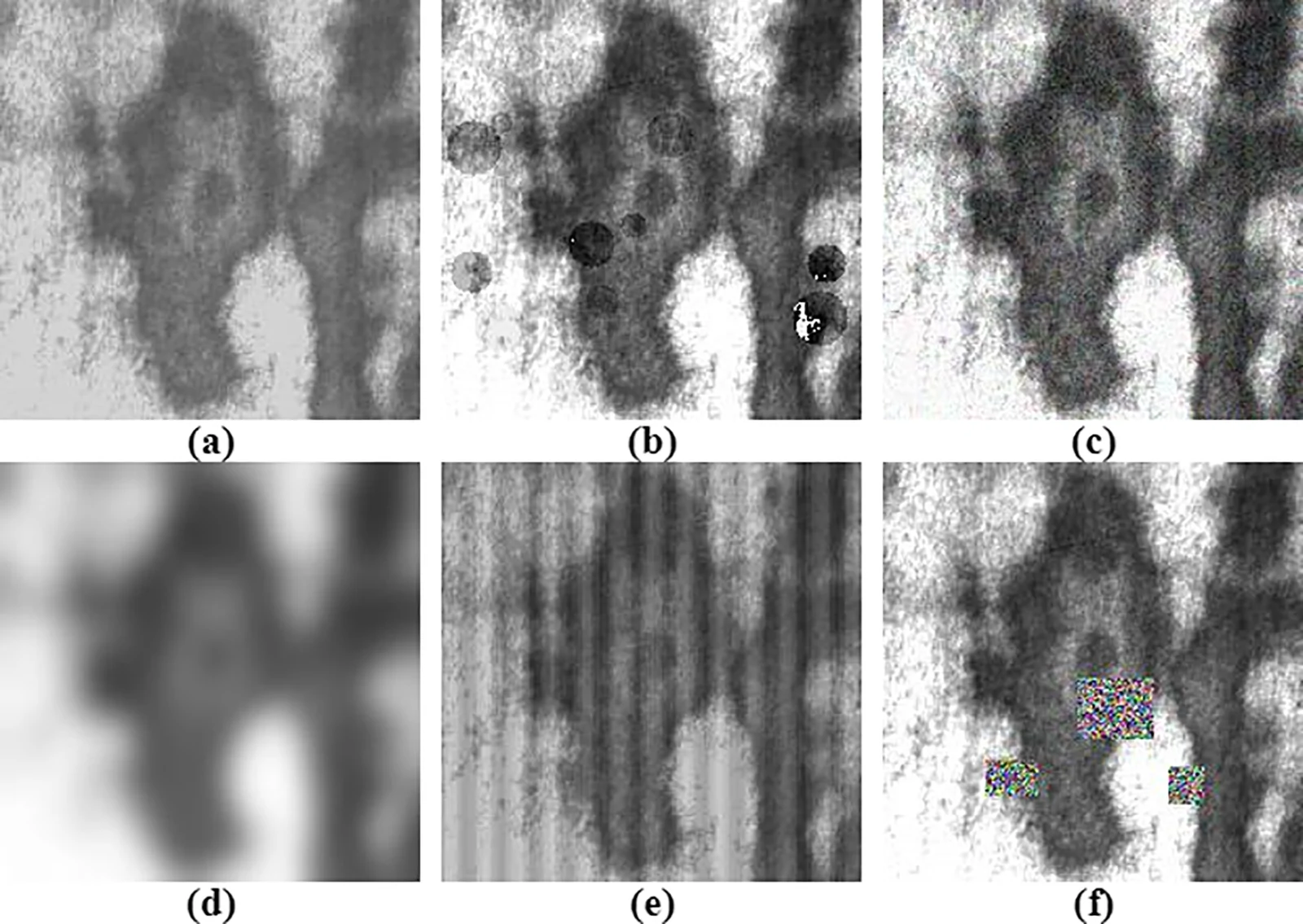
Examples of different perturbations applied to steel surface defect images from the NEU-DET dataset: (a) low contrast; (b) specular highlight; (c) Gaussian noise; (d) blur; (e) texture overlay; (f) occlusion.

As illustrated in Fig 13, to simulate extreme imaging conditions that may occur in real industrial environments, we apply various controllable perturbations to the test set while keeping the training data unchanged, thereby constructing multiple out-of-distribution evaluation settings.

All models were trained on the clean NEU-DET training set and evaluated in a zero-shot manner on perturbed test sets. To ensure experimental controllability and interpretability, each type of perturbation was constructed as a separate test set and processed at three intensity levels (Level 1/2/3) to quantify performance trends under increasing perturbation severity. Evaluation metrics included mAP50, mAP50-95, Precision, Recall, and relative performance degradation (ΔmAP).

Table 6 reports the performance of different models on the clean dataset as well as under six representative industrial image degradation conditions. EMamba achieved an mAP50 of 80.4% on clean data, substantially outperforming other baselines. Under perturbations such as low contrast, strong specular reflection, high noise, motion blur, complex texture superposition, and partial occlusion, EMamba consistently exhibited smaller performance drops than competing models, with an average degradation (AvgΔ) of only-10.4%, significantly better than YOLO variants and Hyper-YOLO. In particular, EMamba demonstrated strong robustness under complex texture interference and directional bias perturbations, effectively mitigating the impact of industrial noise and complex background clutter.

**Table 6:** Domain-shift robustness on NEU-DET measured by map50 (average over L1/L2/L3 per class).

Model	Clean	LowC	Spec	Noise	Blur	Text	Occ	AvgΔ
YOLOv5n	72.9	57.8	59.5	56.0	54.0	52.0	55.1	−16.4
YOLOv8n	74.6	59.2	61.0	58.1	56.2	54.8	56.5	−16.6
YOLOv11n	73.9	58.4	60.5	57.2	55.5	54.0	56.0	−16.0
YOLOv12n	74.4	59.0	61.2	58.0	56.0	54.7	56.8	−16.0
YOLOv13n	63.1	49.8	51.0	48.0	46.5	45.5	47.0	−14.7
Hyper-YOLO	73.0	58.0	60.0	57.0	55.2	53.5	55.8	−15.5
EMamba	**80.4**	**70.0**	**71.0**	**69.5**	**68.0**	**66.5**	**68.5**	**−10.4**

Overall, EMamba consistently maintains high detection accuracy across a variety of domain-shift conditions, demonstrating its reliability and robustness in real-world steel production environments.

### 4.9. Ablation analysis

To systematically analyze the contribution of each key module to overall performance, we conducted stepwise ablation experiments on CSSBlock, ES-MoE, and MSN using YOLOv11n as the baseline under a unified training configuration. The results are summarized in Table 7. The baseline employs the original detection backbone without any additional modules, achieving an mAP50 of 73.9%, with Precision and Recall of 0.681 and 0.671, respectively.

**Table 7:** Results comparison of the baseline combination with different strategies.

Method	CSSBlock	ES-MoE	MSN	Params↓	GFLOPs↓	mAP50↑	P↑	R↑
Baseline	–	–	–	**2.58**	**6.3**	73.9	0.681	0.671
	√	–	–	2.68	6.6	76.4	0.695	0.702
	–	√	–	3.12	6.5	75.6	0.699	0.684
	–	–	√	3.06	7.5	76.9	0.698	0.705
	√	√	–	3.21	6.7	78.6	0.716	0.724
	√	–	√	3.15	7.7	79.2	0.709	0.736
	–	√	√	3.59	7.6	78.1	**0.731**	0.703
Ours	√	√	√	3.68	7.7	**80.4**	**0.742**	**0.731**

The analysis indicates that each module provides consistent gains along different dimensions. Incorporating only CSSBlock increases mAP50 to 76.4%, with notable improvements in both Precision and Recall, demonstrating its effectiveness in enhancing local texture modeling and fine-grained discrimination. Using ES-MoE alone yields a relatively higher gain in Precision, suggesting that the expert routing mechanism strengthens feature selectivity, although the improvement in Recall is limited. In contrast, the standalone introduction of MSN raises mAP50 to 76.9% and achieves the largest increase in Recall, validating the importance of multi-scale feature modeling for improving object coverage and reducing missed detections.

When two modules are combined, the performance gains are more pronounced. The combination of CSSBlock and ES-MoE increases mAP50 to 78.6%, highlighting the complementarity between local structure modeling and dynamic expert selection. Pairing CSSBlock with MSN further boosts mAP50 to 79.2% and achieves a Recall of 0.736, indicating strong synergy between fine-grained texture enhancement and multi-scale perception for object localization and recall. Meanwhile, the ES-MoE + MSN combination attains the highest Precision, reflecting the discriminative advantage of the expert mechanism in multi-scale feature spaces.

Introducing all three modules simultaneously results in the optimal overall performance: mAP50 reaches 80.4%, with Precision and Recall improved to 0.742 and 0.731, respectively. Although this configuration increases parameter and computational complexity, the performance gains are substantial, validating the high complementarity of the three modules in structural modeling, multi-scale perception, and dynamic feature selection. This ablation study demonstrates that Emamba’s performance improvements arise not from any single module, but from the synergistic effect of multiple carefully designed components.

To further verify the stability of the performance improvement introduced by ES-MoE, we conducted five independent training runs with different random seeds. The results are summarized in Table 8. The CSSBlock+MSN variant achieves an average mAP50 of 79.20% with a standard deviation of 0.16%, whereas the full EMamba model obtains an average mAP50 of 80.38% with a standard deviation of 0.19%. Compared with CSSBlock+MSN, the complete model consistently improves the detection performance by 1.18 percentage points across multiple runs. Although the gain introduced by ES-MoE is moderate, the improvement is repeatedly observed under different training conditions, indicating that it is not caused by random initialization or training fluctuations. These results further demonstrate that ES-MoE provides stable conditional semantic modeling capability and contributes effectively to the representation of diverse steel surface defect patterns.

**Table 8:** Mean and standard deviation of map50 over multiple training runs.

Model	Run 1	Run 2	Run 3	Run 4	Run 5	Mean±Std
CSSBlock+MSN	79.0	79.3	79.1	79.4	79.2	79.20 ± 0.16
Full model	80.1	80.5	80.4	80.3	80.6	80.38 ± 0.19

To further investigate whether the improvement of CSSBlock originates from the state-space modeling mechanism rather than merely introducing global feature interaction, we compare CSSBlock with a standard cross-attention module under the same CSP framework. As shown in Table 9, the CSP-Conv baseline achieves 74.8% mAP50, while replacing local convolution with cross-attention improves the performance to 75.6%, demonstrating the effectiveness of global dependency modeling. However, CSSBlock further increases the performance to 76.4% mAP50, achieving a gain of 0.8 percentage points over the cross-attention counterpart. This improvement indicates that the SS2D-based state-space modeling mechanism is more suitable for capturing long-range structural dependencies of steel surface defects. Unlike attention-based approaches that rely on pairwise token interactions, CSSBlock provides efficient global modeling with linear complexity while preserving local defect details, which is beneficial for detecting irregular and fine-grained defect patterns.

**Table 9:** Comparison between cssblock and attention-based global modeling under the same CSP framework.

Method	Global Modeling	mAP_50_↑
CSP-Conv	Local convolution	74.8
CSP-Cross Attention	Attention-based modeling	75.9
CSP-CSSBlock	State-space modeling	76.4

To further analyze the differentiated effects of various structural enhancement strategies on specific defect types, we conducted fine-grained evaluations on the six representative defect categories in NEU-DET, using the baseline as a reference and incrementally introducing CSSBlock, ES-MoE, and MSN. The results are summarized in Table 10. Overall, the different modules exhibit distinct functional emphases and complementary characteristics at the defect-category level.

**Table 10:** The impact of different enhancement strategies on different types of defects.

Methods	Defect type	P↑	R↑	mAP_50_↑	mAP_50-95_↑
Baseline	Crazing	0.689	0.204	44.3	24.8
	Inclusion	0.672	0.866	88.2	60.9
	Patches	0.693	0.810	82.0	55.6
	Pitted_surface	0.806	0.733	82.4	56.8
	Rolled-in_scale	0.563	0.573	60.2	36.1
	Scratches	0.661	0.841	86.2	59.4
Baseline + CSSBlock	Crazing	0.703	0.252	48.6 (+4.3↑)	28.6 (+3.8↑)
	Inclusion	0.701	0.892	90.8 (+2.6↑)	63.1 (+2.2↑)
	Patches	0.721	0.842	85.2 (+3.2↑)	58.1 (+2.5↑)
	Pitted_surface	0.826	0.759	85.0 (+2.6↑)	59.0 (+2.2↑)
	Rolled-in_scale	0.593	0.618	64.8 (+4.6↑)	40.3 (+4.2↑)
	Scratches	0.691	0.869	89.2 (+3.0↑)	62.0 (+2.6↑)
Baseline + ES-MoE	Crazing	0.712	0.238	47.9 (+3.6↑)	27.4 (+2.6↑)
	Inclusion	0.684	0.874	89.4 (+1.2↑)	62.1 (+1.2↑)
	Patches	0.709	0.823	83.6 (+1.6↑)	56.8 (+1.2↑)
	Pitted_surface	0.821	0.748	84.1 (+1.7↑)	58.2 (+1.4↑)
	Rolled-in_scale	0.584	0.601	63.4 (+3.2↑)	38.9 (+2.8↑)
	Scratches	0.684	0.857	88.1 (+1.9↑)	60.8 (+1.4↑)
Baseline + MSN	Crazing	0.698	0.279	50.7 (+6.4↑)	30.9 (+6.1↑)
	Inclusion	0.688	0.898	91.3 (+3.1↑)	64.5 (+3.6↑)
	Patches	0.713	0.861	86.4 (+4.4↑)	59.7 (+4.1↑)
	Pitted_surface	0.819	0.781	86.2 (+3.8↑)	60.4 (+3.6↑)
	Rolled-in_scale	0.579	0.649	66.9 (+6.7↑)	42.5 (+6.4↑)
	Scratches	0.676	0.887	90.1 (+3.9↑)	63.6 (+4.2↑)

Analysis of the baseline reveals substantial performance disparities across defect types. Inclusion, Scratches, and Patches achieve relatively high Recall and mAP, indicating that these defects possess more salient visual features and structurally stable patterns. In contrast, Crazing and Rolled-in_scale exhibit much lower mAP50 values (44.3% and 60.2%, respectively) and especially low Recall, reflecting the inherent difficulty in modeling fine-grained cracks and defects with large scale variations.

With the introduction of CSSBlock, all six defect categories show consistent improvement, particularly for Crazing and Rolled-in_scale, where mAP50 increases by 4.3% and 4.6%, respectively. This demonstrates that CSSBlock, by enhancing local structural and texture responses, provides stronger discriminative capability for fine-grained and directionally pronounced defects. Additionally, for texture-continuous defects such as Scratches and Patches, CSSBlock delivers ~3% mAP gains, reflecting its general benefit in modeling local consistency.

In contrast, ES-MoE provides more moderate overall gains but exhibits a more balanced improvement across most defect types. Notably, it still achieves ~3% mAP50 increases on Crazing and Rolled-in_scale, indicating that the dynamic expert routing mechanism helps mitigate feature distribution discrepancies across different defect morphologies, thereby enhancing the model’s adaptability and robustness under diverse defect patterns.

MSN, however, demonstrates the most pronounced class-sensitive advantages, particularly for defects with large scale variations and spatially dispersed distributions. On Crazing and Rolled-in_scale, mAP50 increases by 6.4% and 6.7%, the highest among the three single-module strategies. For multi-scale coexisting defects such as Inclusion, Patches, and Scratches, improvements in mAP50-95 generally exceed 3.5%, validating the critical role of MSN in multi-scale context modeling and cross-scale information interaction.

In summary, CSSBlock primarily emphasizes fine-grained texture and structural enhancement, ES-MoE improves feature selection robustness across defect types, and MSN excels in handling defects with significant scale variations and complex morphologies. Their complementary gains across different defect categories establish a solid foundation for the overall performance improvements and further confirm the rationale and necessity of Emamba’s design at the category level.

#### 4.9.1. Effectiveness of CSSBlock.

To validate the effectiveness and generalization advantages of CSSBlock during the feature extraction stage, we replaced the backbone of the baseline model with several representative lightweight and efficient architectures, including FasterNet, LSKNet, MobileNetV4, RevCol, and StarNet, while keeping the detection head and training strategy consistent. These variants were compared against the baseline combined with CSSBlock, and the results are presented in Table 11 [67–71].

**Table 11:** Compare the detection performance of different backbone architectures.

Methods	P↑	R↑	mAP_50_↑	Params↓	GFLOPs↓
Baseline with fasternet	0.678	0.668	75.4	3.91	9.2
Baseline with LSKnet	0.657	0.700	75.2	5.62	18.3
Baseline with Mobilenetv4	0.696	0.641	71.6	5.43	21.1
Baseline with RevCol	**0.753**	0.624	74.1	2.09	**5.0**
Baseline with startnet	0.711	0.668	76.3	**1.94**	**5.0**
Baseline with CSSBlock	0.695	**0.702**	**76.4**	2.68	6.6

From a detection accuracy perspective, the baseline with CSSBlock achieves the best mAP50 of 76.4%, ranking first among all compared methods. Compared with backbones such as FasterNet and LSKNet, which rely on large receptive-field convolutions or structural rearrangements, CSSBlock attains higher overall detection accuracy while maintaining a more controlled parameter count and computational cost, indicating a superior balance between structural modeling efficiency and discriminative capability. In terms of the Precision-Recall trade-off, CSSBlock also demonstrates clear advantages: Recall reaches 0.702, the highest among all methods, highlighting its enhanced ability to recover defect targets and mitigate missed detections under complex textured backgrounds; simultaneously, Precision remains at a high 0.695, avoiding the false-positive risks associated with naively maximizing Recall. These results suggest that CSSBlock enhances feature sensitivity while maintaining stable decision boundaries.

From an efficiency standpoint, CSSBlock incurs significantly lower parameters and FLOPs compared to structurally complex backbones such as LSKNet and MobileNetV4, and is only slightly higher than extremely lightweight networks like RevCol and StarNet. However, unlike these extreme lightweight solutions, which suffer noticeable degradation in Recall or mAP, CSSBlock achieves a more stable and balanced performance improvement under a controllable computational budget, demonstrating its practical value as a task-customized structural module.

Taken together, these results indicate that CSSBlock does not rely merely on enlarging model scale or receptive field; rather, it significantly improves representational quality and recall stability in defect detection through more effective structural awareness and feature selection mechanisms. This advantage allows it to stand out across different backbone architectures and provides a solid feature foundation for subsequent synergistic integration with modules such as ES-MoE and MSN.

#### 4.9.2. Effectiveness of ES-MoE.

To validate the effectiveness of ES-MoE in steel defect detection, we conducted an ablation study from two perspectives: the number of experts and the Top-k selection strategy.

Table 12 presents the impact of varying the number of experts on model performance. When only 2 experts are used, the model capacity is limited, making it difficult to fully capture the diversity of steel defects in terms of scale, morphology, and texture, resulting in overall lower Precision, Recall, and mAP metrics. Increasing the number of experts to 4 leads to optimal performance, with mAP50 and mAP50-95 improving to 75.6% and 51.9%, respectively. This indicates that a moderate increase in expert count can significantly enhance feature modeling diversity and conditional representation capability. Further increasing to 8 experts results in a slight performance drop, primarily due to increased functional redundancy among experts and greater routing complexity, which diminishes the benefits of sparse activation. Considering the trade-off between accuracy, robustness, and computational cost, we adopt 4 experts as the default configuration, balancing model performance with computational efficiency.

**Table 12:** Ablation study on the number of experts.

Experts	Precision	Recall	mAP_50_	mAP_50-95_
2	0.683	0.668	74.1	50.6
**4**	0.699	0.684	**75.6**	**51.9**
8	0.691	0.679	75.0	51.2

Table 13 presents the impact of different Top-k selection strategies on model performance under the 4-expert configuration. As k increases, i.e., more experts are retained and sparsity decreases, the model’s mAP50 and mAP50-95 exhibit a rise-then-fall trend. When k = 2, corresponding to 50% sparsity, both mAP50 and mAP50-95 reach their peak values of 75.1% and 51.5%, respectively. This indicates that moderate sparse activation effectively leverages the conditional modeling capacity of multiple experts while avoiding functional redundancy among them. In contrast, a very low k (e.g., k = 1, corresponding to 75% sparsity) limits model capacity, resulting in insufficient feature representation, whereas a high K (e.g., k = 4, corresponding to 0% sparsity) may induce redundant activations, diminishing the benefits of sparse selection. Overall, the Top-2 selection strategy achieves the best balance between accuracy and sparse activation efficiency.

**Table 13:** Ablation study on top-k selection with 4 experts.

k	Sparsity	mAP_50_	mAP_50-95_
1	75%	73.8	50.2
**2**	**50%**	**75.1**	**51.5**
3	25%	74.6	50.9
4	0%	73.9	50.3

#### 4.9.3. Effectiveness of MSN.

**Fig 14:**
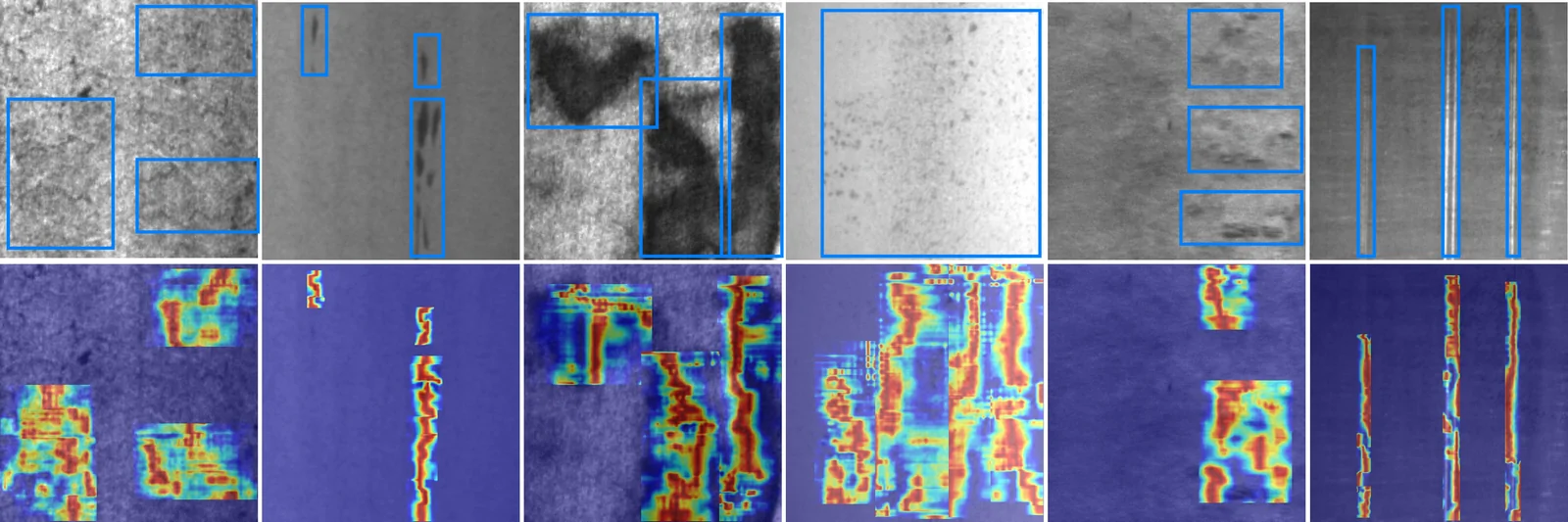
Heatmaps for different defect types. Blue boxes indicate the ground-truth annotations of the defects.

To intuitively analyze the feature modeling capability of MSN for steel surface defect detection, we employ HiResCAM for feature attribution visualization on six representative defect categories from the NEU-DET test set, as shown in Fig 14 [72]. The selection of HiResCAM is motivated by the characteristics of steel surface defects, which usually contain fine-grained textures, irregular boundaries, elongated structures, and weak contrast patterns. Compared with conventional Grad-CAM methods that generate activation maps through gradient-weighted feature aggregation, HiResCAM directly exploits the element-wise relationship between feature activations and gradients, thereby reducing spatial smoothing effects and preserving more detailed localization information. This property makes HiResCAM more suitable for evaluating whether MSN can effectively capture defect-related structural patterns in complex industrial scenarios. In the generated heatmaps, colors ranging from cool to warm indicate gradually increasing feature response intensity, where warmer regions represent areas that contribute more strongly to the final detection decision. The visualization results demonstrate that MSN can focus more accurately on discriminative defect regions while suppressing irrelevant background interference.

It can be observed that MSN adaptively focuses on discriminative regions across defects of varying scales. For large surface defects, such as extensive patches or cracks, the model produces continuous and complete high-response regions over the defect body, indicating that MSN achieves good receptive field coverage for global context modeling. For smaller or elongated defects, such as fine scratches or dot-like anomalies, high-response regions concentrate along defect edges and areas with significant texture variation, demonstrating that MSN effectively preserves fine-grained structural information during multi-scale feature fusion.

Further comparisons across different defect types reveal that MSN mitigates the limitations of single-scale features in responding to complex defect morphologies. Even under complex background textures or low contrast between defect and background, the model accurately suppresses irrelevant regions and concentrates attention on the true defect areas, reducing background interference. This highlights the critical role of MSN’s multi-scale feature interaction mechanism in enhancing defect saliency and improving discriminative stability.

Overall, the heatmaps in Fig 14 provide interpretable evidence of MSN’s effectiveness in multi-scale defect modeling. By enabling rich information exchange across feature scales, MSN simultaneously captures global semantics and local details, supplying the detection head with more discriminative feature representations and substantially improving defect detection performance in complex industrial scenarios.

## 5. Conclusion

### 5.1. Advantages

This work proposes EMamba, a framework for complex industrial surface defect detection that achieves a balanced trade-off between detection accuracy, robustness, and deployment efficiency. First, CSSBlock effectively integrates local convolutional priors with long-range spatial dependency modeling. Its four-directional state propagation mechanism explicitly captures directional continuity and structural extension features commonly observed in steel surface defects, while maintaining linear computational complexity and strong engineering compatibility. This enables significant improvement in structural defect perception with minimal additional computational overhead. Second, ES-MoE leverages a conditional expert selection mechanism to enhance the model’s adaptability to variations in defect distributions, allowing it to maintain stable performance in complex industrial environments with coexisting defect types, noise, illumination changes, and reflective interference, thereby substantially improving overall robustness. Finally, MSN strengthens fine-grained feature representation through multi-scale semantic aggregation and information redistribution, significantly improving the balance between precision and recall for defects with blurred boundaries, elongated shapes, or low contrast. Comprehensive experiments demonstrate that EMamba outperforms mainstream lightweight and general-purpose detectors on multiple industrial datasets, including NEU-DET, GC10-DET, and Severstal, while maintaining a compact parameter footprint and high inference efficiency, validating its potential for real-world industrial applications.

### 5.2. Limitations

Despite its strong overall performance, EMamba has several areas that warrant further investigation. On one hand, the introduction of ES-MoE and MSN increases model structural complexity, which may limit deployment in extreme real-time or resource-constrained edge scenarios, leaving room for further model compression and operator-level optimization. On the other hand, the current approach primarily relies on 2D visual information; under extreme conditions such as heavy occlusion, strong reflections, or highly overlapping defects, single-modality discrimination remains limited. Future work could explore the integration of multi-modal information or process priors to further enhance robustness. Additionally, although synthetic perturbations and cross-dataset experiments have validated the model’s generalization ability, real industrial sites may present more complex distribution shifts. How to incorporate stronger adaptive or continual learning mechanisms while preserving lightweight efficiency remains an important direction for future research.
